# Identification of microRNA 885-5p as a novel regulator of tumor metastasis by targeting CPEB2 in colorectal cancer

**DOI:** 10.18632/oncotarget.15844

**Published:** 2017-03-02

**Authors:** Colin Siu-Chi Lam, Lui Ng, Ariel Ka-Man Chow, Timothy Ming-Hun Wan, Simon Yau, Nathan Shiu-Man Cheng, Sunny Kit-Man Wong, Johnny Hon-Wai Man, Oswens Siu-Hung Lo, Dominic Chi-Chung Foo, Jensen Tung-Chung Poon, Roberta Wen-Chi Pang, Wai-Lun Law

**Affiliations:** ^1^ Division of Colorectal Surgery, Department of Surgery, Li Ka Shing Faculty of Medicine, The University of Hong Kong, Hong Kong; ^2^ Centre for Cancer Research, Li Ka Shing Faculty of Medicine, The University of Hong Kong, Hong Kong

**Keywords:** CRC, liver metastasis, miR-885-5p, EMT, CPEB2

## Abstract

Colorectal cancer is the third most common cancer in the world and liver is the most frequent site of distant metastasis with poor prognosis. The aim of this study is to investigate microRNAs leading to liver metastasis. We applied microarray analysis and quantitative PCR to identify and validate dysregulated miRNAs in liver metastases when compared to primary CRCs. Functional significance and the underlying molecular mechanism of selected miRNA was demonstrated by a series of *in vitro* and *in vivo* assays. Our microarray analysis and subsequent quantitative PCR validation revealed that miR-885-5p was strongly up-regulated in liver metastases and in CRC cell-lines derived from distant metastases. Overexpression of miR-885-5p significantly induced cell migration, cell invasion, formation of stress fibre *in vitro* and development of liver and lung metastases *in vivo*. MiR-885-5p induced metastatic potential of CRC by repressing cytoplasmic polyadenylation element binding protein 2 transcription through directly binding to two binding sites on its 3′ untranslated region, and consequently led to up-regulation of TWIST1 and hence epithelial-mesenchymal transition. Our findings demonstrated the overexpression of miR-885-5p in liver metastasis and its roles in inducing CRC metastasis, potentiating development of miR-885-5p inhibitor to treat advanced CRC in the future.

## INTRODUCTION

Colorectal cancer (CRC) is third most common malignancy and the third leading cause of cancer death in the United States and Hong Kong [1]. The patients with metastasis have a higher mortality rate than primary tumor development alone [2]. Liver is the most common site of distant metastasis with poor prognosis. Recently, microRNAs (miRNAs) have been identified as important molecules in regulating protein expression for metastasis [3, 4]. Understanding the biological mechanism of miRNAs in regulating liver metastasis is beneficial for developing new therapies.

Metastasis formation is a complex multi-step process which is regulated by different signaling pathways and cell adhesion molecules (CAMs) [5, 6]. Epithelial-mesenchymal transition (EMT), which is characterized by the cell reprogramming and transition from epithelial phenotype to mesenchymal phenotype, is a pivotal process that affects the tumor cell metastasis by altering cell-cell contact and cell-extracellular matrix (ECM) interactions [7, 8]. Tumors derived from epithelial cells can become more motile and invasive by acquiring characteristics of mesenchymal cells [9]. E-cadherin, which is expressed in epithelial cells and promotes cell-cell adhesion, is one of the decreased epithelial markers during EMT while N-cadherin, Vimentin and Snail are increased mesenchymal markers [10]. Down-regulation of E-cadherin is mediated by transcription factors such as Snail and Slug, so up-regulation of Snail has also been implicated as an EMT marker for metastatic cancers [11, 12].

A recent study demonstrated the critical role for miRNAs in EMT, a phenotypic change undergone by altered gene expression patterns that is theorized to have central roles in metastatic progression [13]. MiRNAs are a class of small (∼22-nucleotide) non-coding RNAs that play a critical role in cellular proliferation, angiogenesis, differentiation and apoptosis by regulating gene expression via either specific post-translational repression or mRNA cleavage targeting [14, 15]. Regulation of miRNAs contributes to the etiology and process of oncogenesis through targeting of oncogenes or tumor suppresser genes [16–18]. Thus, miRNAs may be used as potential biomarkers for cancer diagnosis and prognosis [19].

Rho-GTPase family, RhoA, ROCK (Rho-associated coiled coil-containing protein kinase) and cdc42, participate in tumor cell invasion and migration through modulating the organization of stress fibers and focal adhesions [20]. MiRNAs are important for tissue morphogenesis by regulating cytoskeletal remodelling and phenotypic switching [21]. Besides, cell extravasation through the endothelium is an important event for tumor metastasis [6]. TWIST1 is a basic helix-loop-helix transcription factor which contributes to metastasis in many human cancers through promoting EMT pathway [22, 23]. Cytoplasmic polyadenylation was a mechanism that regulates the expression of gene by controlling poly(A) tail length in mammalian cells [24]. Cytoplasmic polyadenylation element binding protein (CPEB), which is the mediator of cytoplasmic polyadenylation, binds to the cytoplasmic polyadenylation element (CPE) in the 3′UTR of mRNA [25]. *TWIST1* expression was regulated by cytoplasmic polyadenylation element binding protein 2 (CPEB2) post-transcriptionally in CPE-dependent manner [26].

Since tumor cells have differential miRNA expression, it is critical to understand the mechanism behind this which may be beneficial for treatment [27, 28]. Thus, the difference between primary colon cancer and liver metastasis was investigated in this study to identify the novel factors that participate in liver metastasis for CRC.

## RESULTS

### miR-885-5p was significantly up-regulated in liver metastasis and metastatic CRC cell lines

**Figure 1:**
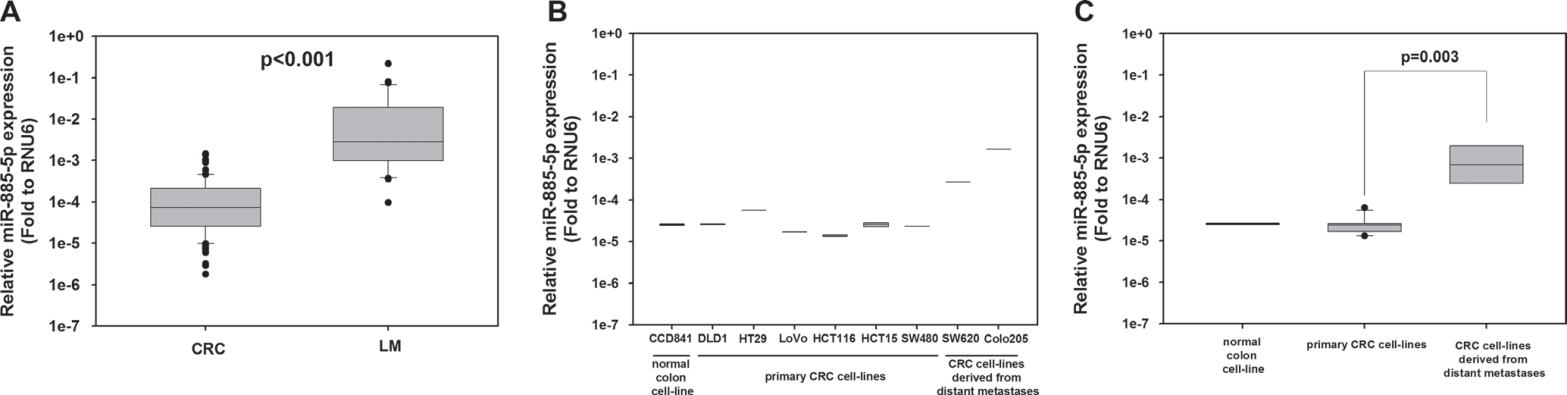
High miR-885-5p level is associated with distant metastasis in CRC patient samples and cell-lines (**A**) Expression of miR-885-5p in 104 CRC and 39 liver metastasis (LM) specimens was detected by qRT-PCR. The relative miR-885-5p level in liver metastases (median = 0.00284) was significantly higher than that in CRC (median: 0.0000734; *P* < 0.001 by Mann-Whitney Rank Sum test). (**B**) Expression of miR-885-5p in normal colon cell-line CCD-841-CoN (CCD841), primary CRC cell-lines (DLD1, HT29, LoVo, HCT116, HCT15 and SW480) and CRC cell-lines derived from distant metastases (SW620 and Colo205) was detected by qRT-PCR. (**C**) Comparison of miR-885-5p level between grouped primary CRC cell-lines and grouped CRC cell-lines derived from distant metastases. The relative expression of miR-885-5p was calculated by the 2−ΔCt method: 2^−(Ct of miR-885-5p – Ct of RNU6)^. Data shown is the mean value (± SEM) of triplicates from three independent experiments.

To identify miRNAs associated with liver metastasis, we performed TaqMan^®^ Low Density Array Human MicroRNA Panel with 754 human unique miRNAs (Sanger miRBase v16) to investigate the miRNA expression profiles of five human primary CRCs and five liver metastasis tumors. Among the miRNAs screened, miR-885-5p was highly up-regulated (∼28.5-fold change, *P* = 0.036) in liver metastasis tumors when compared with primary CRCs (Supplementary Table 1). To confirm the microarray findings, qPCR was used to detect miR-885-5p level in a further 104 primary CRC and 39 liver metastasis tissues. As shown in Figure 1A, the median relative miR-885-5p level in liver metastasis samples (0.00284) was significantly higher than the level in primary CRCs (0.0000734; *P* < 0.001). More importantly, among these, there were two pairs of simultaneous CRC and liver metastasis samples from the same patients. Both of them showed much higher relative miR-885-5p level in their liver metastasis tumors (0.0111 and 0.0629) when compared with the primary CRC (0.0000486 and 0.000180, respectively).

We also determined the expression of miR-885-5p among normal human intestinal epithelial cell line (CCD-841-CoN), primary CRC cell-lines (HCT116, SW480, HT-29, HCT-15 and DLD-1) and cell lines derived from distant metastases of CRC (SW620 and Colo205) (Figure 1B). Comparing SW620 and SW480, which originated from the metastatic site and primary site of the same patient, respectively, miR-885-5p level was around 8-fold higher in SW620. The expression level of miR-885-5p was significantly higher in grouped CRC cell-lines derived from metastatic site when compared with the grouped primary CRC cell-lines (*P* = 0.003; Figure 1C). For downstream functional experiments, primary CRC cell-lines HCT116 and DLD1 which expressed relatively lower level of miR-885-5p were used in miR-885-5p overexpression experiments. On the other hand, SW620 and Colo205 which expressed higher level of miR-885-5p were used in miR-885-5p repression experiments.

### miR-885-5p expression induced CRC cell migration and invasion through activation of epithelial-mesenchymal transition (EMT) pathway

**Figure 2:**
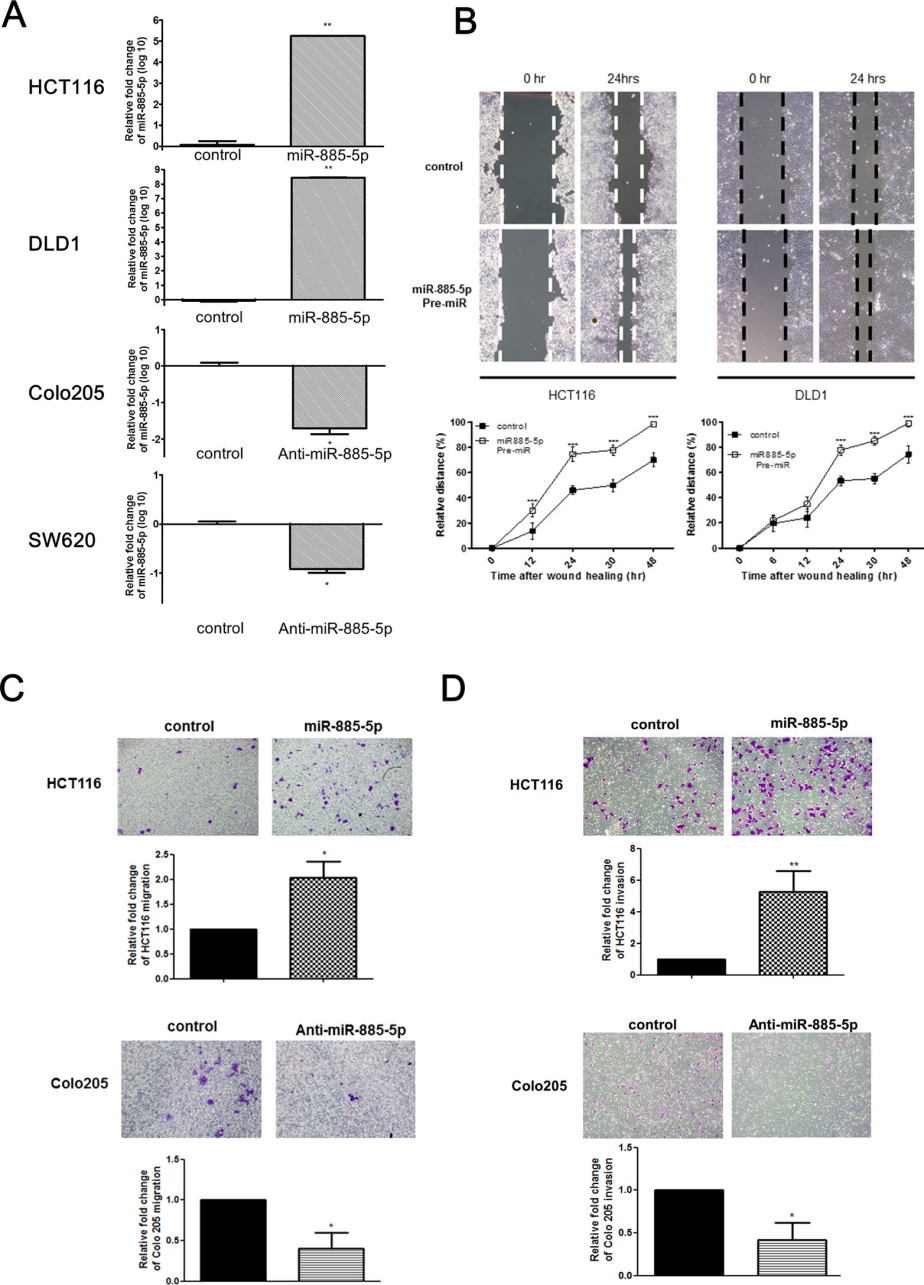
miR-885-5p enhances CRC cells invasive and migratory capacity (**A**) The relative expression of miR-885-5p in miR-885-5p overexpressed or control transfected cells (HCT116 and DLD1) and miR-885-5p repressed or control transfected cells (Colo205 and SW620). (**B**) Wound healing experiment was performed using HCT116 and DLD1 cells transfected with either negative control or Pre-miR miR-885-5p. Representative images of wound were at time 0 hr and 24 hr after monolayer wounding were shown (upper panel). The distance of cell migration was measured every 12 hours and relative distance % was calculated by the formula (initial wound distance –wound distance at time point)/ intial wound distance × 100%. The distance of cell migration was higher in cells transfected with miR-885-5p in both HCT116 and DLD1 cell-lines (lower panel; ***P < 0.001 by student's *t*-test). Data is expressed as mean values (± SEM) of triplicate measurements from three independent experiments. (**C** and **D**) Transwell cell migration (C) and invasion assays (D) were performed using HCT116 cells transfected with either control or miR-885-5p (upper panel) and Colo205 cells transfected with either negative control or anti-miR-885-5p inhibitor (lower panel). Cells migrated or invaded were stained with 0.2% crystal violet and the number was counted under microscope. The number of cells migrated or invaded was significantly higher in HCT116 cells transfected with miR-885-5p than that with control, and significantly lower in Colo205 cells transfected with anti-miR-885-5p inhibitor than with negative control (*P < 0.05, student's *t*-test). Data is expressed as mean values (± SEM) of five measurements in three independent experiments.

To examine whether miR-885-5p functionally contributed to enhanced metastatic potential of CRC cells, miR-885-5p was ectopically expressed or repressed in CRC cell lines and the effect on the migratory capacities (invasion and migration) was studied (Figure 2A). HCT116 and DLD1 cell-lines were transiently transfected with control or miR-885-5p precursors. miR-885-5p overexpressing cells migrated at a higher rate than negative control as shown by the wound-healing assays (Figure 2B). Figure 2B lower panel shows the quantified distance between the migrated cells at different time points. Similar results were observed when transfectants were subjected to migration chamber assays *in vitro* (Figure 2C). miR-885-5p overexpressing cells have greater migratory capacity (∼2 fold) than the transfected negative control. On the other hand, high miR-885-5p expressing cells Colo205 were transiently transfected with miR-885-5p inhibitor (Anti-miR) to study the effect of miR-885-5p repression. Migration rate of cells with miR-885-5p inhibition was significantly impaired in comparison to the control which was shown by the migration chamber assays (Figure 2C).

We also demonstrated the causative role of miR-885-5p expression and invasive phenotypes of CRC cells. Overexpression of miR-885-5p substantially increased the invasive properties of CRC cells as shown from the transwell matrigel™ invasion chambers assay (Figure 2D). The cell invasion result was quantified with relative fold change to the mimic control. Invasion assay demonstrated that miR-885-5p overexpressed cells showed around five times more number of invaded cells when compared with the control transfected cells. On the other hand, miR-885-5p inhibition in Colo205 cells by transient transfection of anti-miR miR-885-5p reduced their invasive capacities when compared with negative control in the invasion chamber assay (Figure 2D).

**Figure 3:**
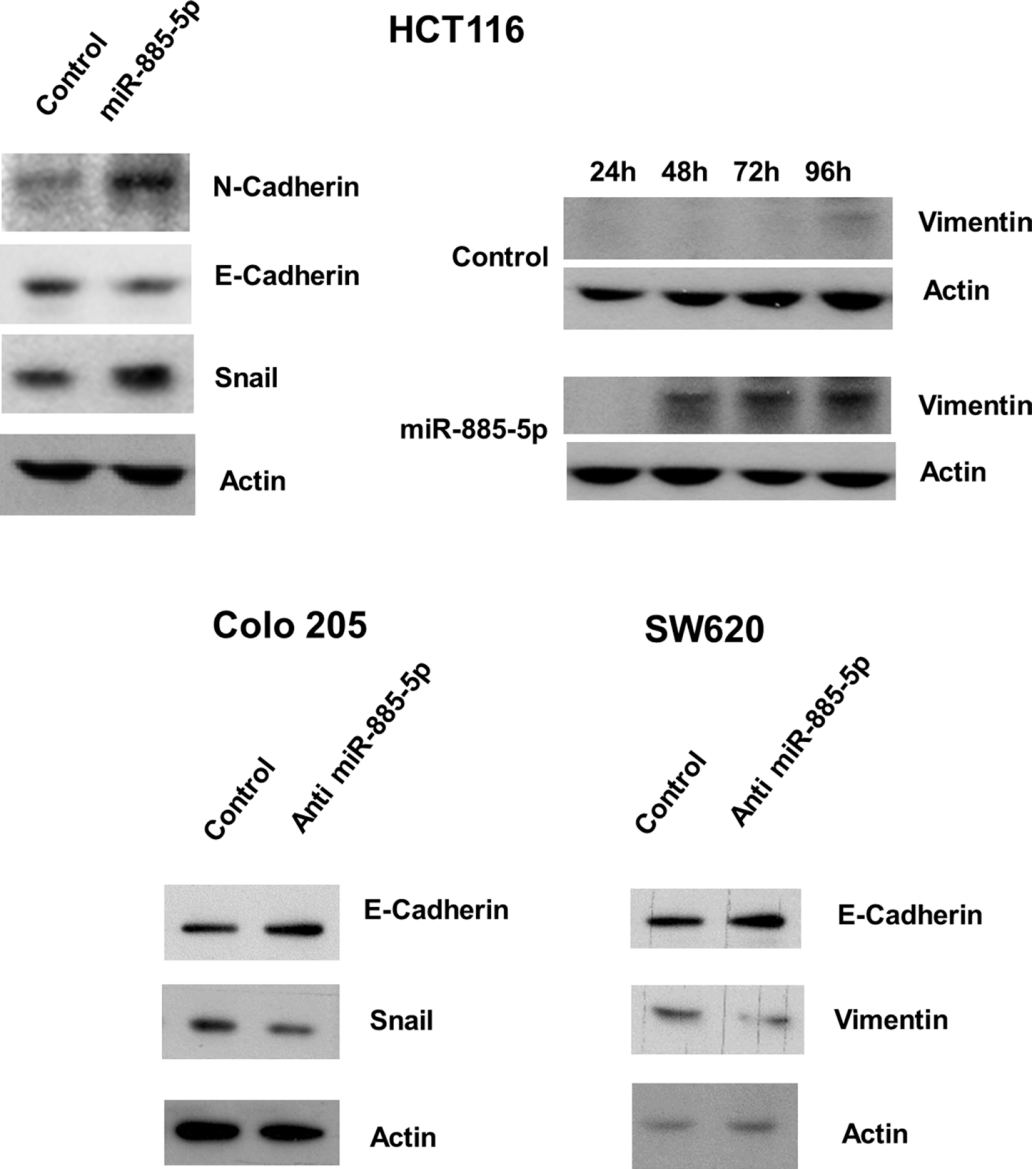
Effect of miR-885-5p on epithelial-mesenchymal transition proteins The effects of miR-885-5p overexpression or inhibition on protein level of epithelial mesenchymal transition pathway were detected by western blotting. Upper panel: miR-885-5p overexpression in HCT116 cells induced N-cadherin and Snail expression and repressed E-cadherin level. The expression of Vimentin at different time points in HCT116 cells was determined in HCT116 cells. Vimentin was weakly expressed in mimic control transfected cells after 96 hours whereas its expression was strongly induced in miR-885-5p overexpressed cells after 48 hours and further induced after 72 and 96 hours. Lower panel: miR-885-5p repression in Colo 205 and SW620 cells induced E-cadherin level whereas repressed Snail in Colo 205 cells and Vimentin in SW620 cells. Total β-actin was used as loading control. Images shown were representative results from at least three independent experiments.

We investigated whether ectopic expression of miR-885-5p alter the EMT pathway of CRC cells, which is an important process for inititation of cancer metastasis. Western blotting revealed that overexpression of miR-885-5p led to repression of E-cadherin and concomitant up-regulation of N-cadherin and Snail (Figure 3). Moreover, Vimentin expression level was increased with time in Pre-miR transfectants. On the other hand, knockdown of miR-885-5p led to enhanced expression of E-cadherin, as well as a concordant decrease in Snail and Vimentin expression in Colo 205 and SW620 respectively. These results suggested that miR-885-5p induced metastatic capacity of CRC cells through activating the EMT pathway.

### Expression of miR-885-5p enhanced stress fiber formation through cytoskeleton rearrangement

**Figure 4:**
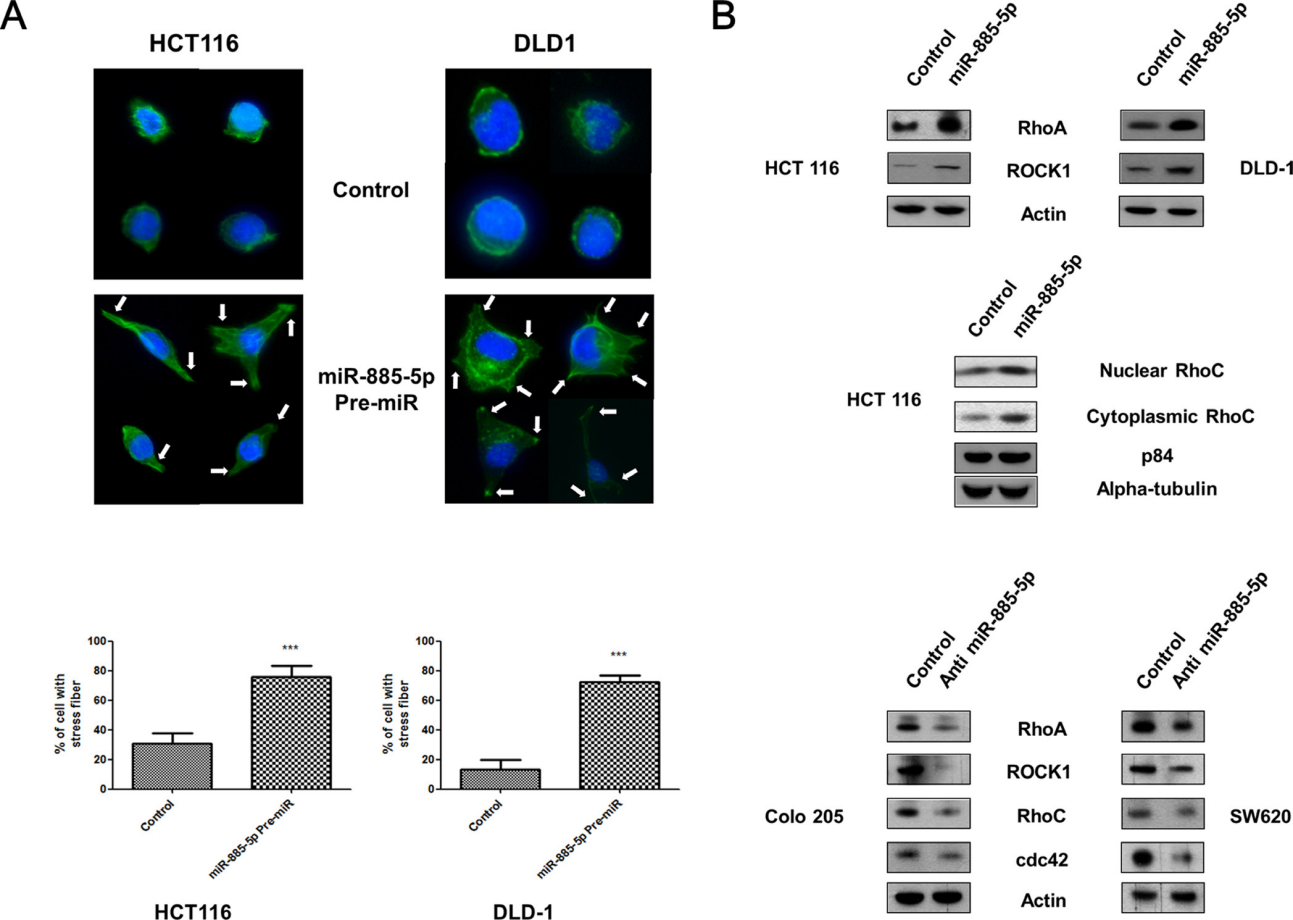
Effect of miR-885-5p on formation of cell protrusions (**A**) Upper panel: HCT 116 and DLD-1 cells transfected with negative control or miR-885-5p were stained with phalloidin and DAPI. Representative micrographs showing formation of cell protrusions (indicated by arrows) and signals from F-actin (green) and nucleus (blue). Scale bar: 50 μm. Lower panel: Bar graph showing percentage of cells with cell protrusion formation in five random area. Data is expressed as the mean (± SEM) of three independent experiments. (***P < 0.001, student's *t*-test). (**B**) Analysis of endogenous levels of Rho-GTPase families by immunoblotting in CRC cell lines transiently transfected with pre-miR-885-5p, negative control or anti-miR-885-5p for 48 hours. Total β-actin was used as a loading control.

We analysed the effect of miR-885-5p on actin stress fiber formation. As shown in Figure 4A, co-staining showed the formation of cell protrusion (indicated by the arrows) was enhanced in miR-885-5p Pre-miR transfectants as compared with actin cortical ring which was observed in control transfectants. Our results also showed that the stress fiber formation of overexpressing miR-885-5p transfectants was increased to ∼80% as compared with ∼20% of the negative control transfectants. Since the cell elasticity is directly influenced by the intracellular actin, over-expressing miR-885-5p clearly demonstrated higher quantities of actin stress fibers formation when compared with the cortical ring in negative control.

De-regulation of Rho family small GTPases have been widely reported to be accounted for cell protrusions and actin stress fiber formation in invasive cancer cells [29]. The effect of miR-885-5p expression on levels of Rho family small GTPases, RhoA, RhoC, ROCK1 and cdc42, was detected by western blotting (Figure 4B). Higher RhoA and ROCK1 expression was exhibited in Pre-miR transfectants. In addition, nuclear and cytooplasmic expression of RhoC was induced by miR-885-5p overexpression. In contrast, the expression of Rho family small GTPases were inhibited in anti-miR transfectants. These results suggested that miR-885-5p induced the actin filaments reorganization through up-regulation of the Rho family small GTPases, leading to the formation of motility structures at the leading edge of cells.

### miR-885-5p down-regulated CPEB2 by directly binding to CPEB2 3′UTR

**Figure 5:**
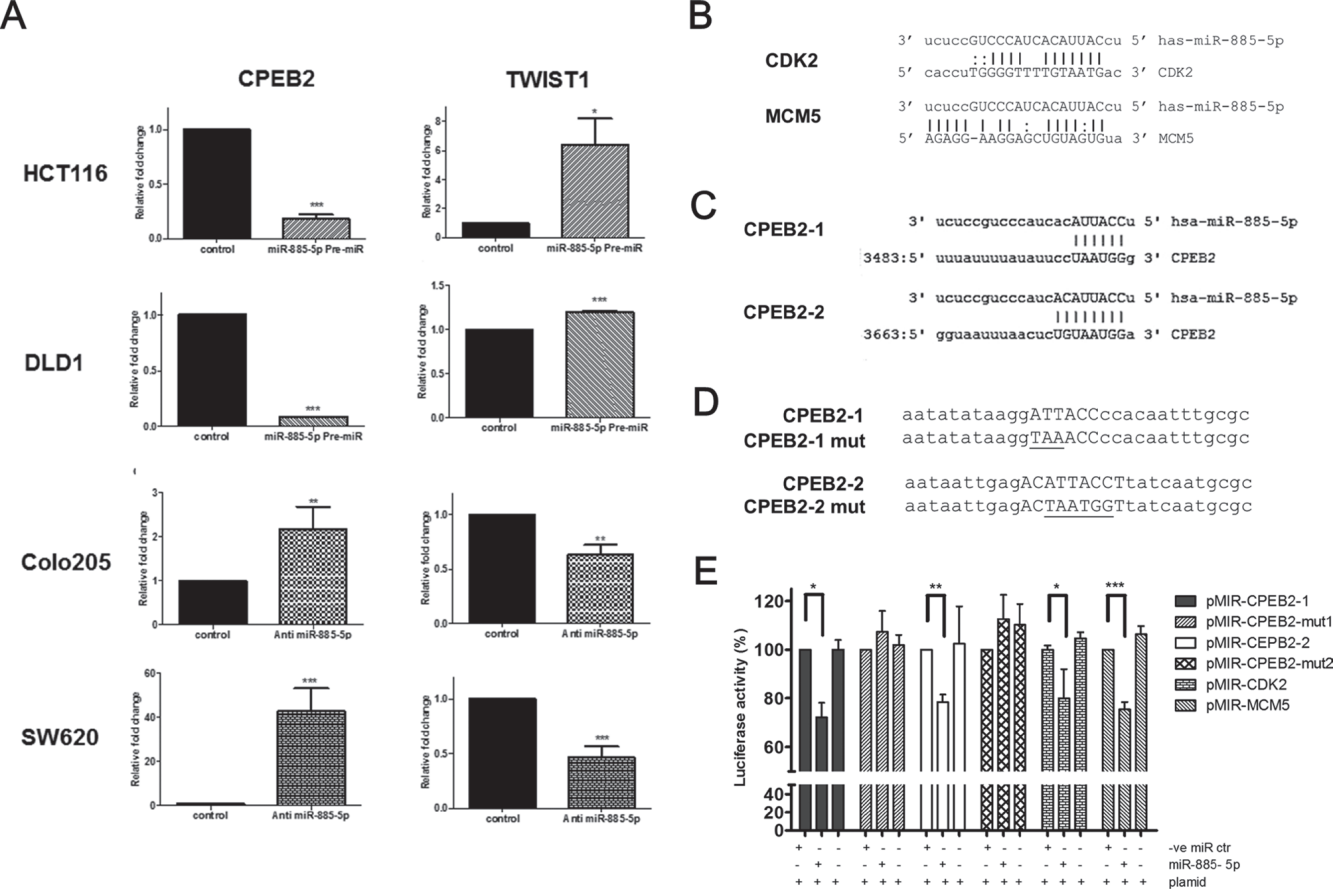
Identification of CPEB2 as a target gene of miR-885-5p (**A**) qPCR was applied to detect the mRNA level of CPEB2 and TWIST1 in HCT116 and DLD1 cells overexpressed with control or miR-885-5p, as well as in Colo205 and SW620 cells transfected with negative control or anti-miR-885-5p inhibitor. Data is expressed as the mean (± SEM) of three independent experiments. (***P < 0.001, **P < 0.01, *P < 0.05, student's *t*-test). (**B**) Reported binding sites of miR-885-5p on CDK2 and MCM5 gene which were used as positive control in our luciferase reporter experiments. (**C**) Two potential binding sites of miR-885-5p (CPEB2-1 and CPEB2-2) were predicted in the 3′UTR region of CPEB2 gene. (**D**) Designs of CPEB2-1 and CPEB2-2 mutants for luciferase reporter assay. *Upper case*: Potential binding sites of miR-885-5p; *Underline*: mutated nucleotides. (**E**) HCT116 cells were transiently co-transfected with miRNA control or miR-885-5p and reporter construct containing miR-885-5p binding sites or mutants of CPEB2 (CPEB2-1 or CPEB2-2). CDK2 and MCM5 are included as positive controls. Firefly luciferase values were normalized by Renilla activity, and results were expressed as the percentage of luciferase activity of each co-transfection with reference to the luciferase activity of the same reporter construct with miRNA negative control only. Data is expressed as the mean (±SEM) of three independent experiments. (***P < 0.001, **P < 0.01, *P < 0.05, student's *t*-test).

We hypothesized that miR-885-5p activated EMT through its potential target gene CPEB2, which is one of the negative regulators of *TWIST1* expression in a sequence-specific manner [26]. Our results showed that CPEB2 was significantly down-regulated in the cells overexpressing miR-885-5p and was conversely up-regulated in anti-miR transfectants, whereas *TWIST1* was conversely expressed with CPEB2 (Figure 5A).

Dual luciferase reporter assays were performed in HCT116 cells to test whether miR-885-5p directly target the CPEB2 3′-UTR. Reporter plasmids containing CDK2 or MCM5 3′-UTR binding sites were included as positive controls [30] (Figure 5B). Two potential target binding sites of miR-885-5p in CPEB2 3′-untranslated region, namely CPEB2-1 and CPEB2-2, were predicted by miRNA database (www.microrna.org) [31] (Figure 5C). Two oligonucleotides containing the CPEC2-1 and CPEB2-2 sequences were cloned into pMIR reporter vector. In addition, reporter constructs containing mutated CPEC2-1 and CPEB2-2 sequences were prepared (Figure 5D). The relative firefly luciferase activities of positive controls were inhibited by around 25% in the presence of miR-885-5p pre-miR co-transfectants (Figure 5E), indicating our system demonstrated the regulation of miR-885-5p on its target genes. Relative firefly luciferase activities of reporter plasmids containing CPEB2-1 or CPEB2-2 3′-UTR binding sites were inhibited by 32–38% in the presence of miR-885-5p Pre-miR but not mimic control nor plasmid only. More importantly, luciferase activity was not affected by miR-885-5p co-transfection when the 3′UTR sequences of the CPEB2-1 and CPEB2-2 reporter plasmids were mutated. These findings showed that miR-885-5p directly targeted the two predicted miR-885-5p binding sites in the CPEB2 3′UTR.

### Role of miR-885-5p in the development of metastasis of CRC *in vivo*

**Figure 6:**
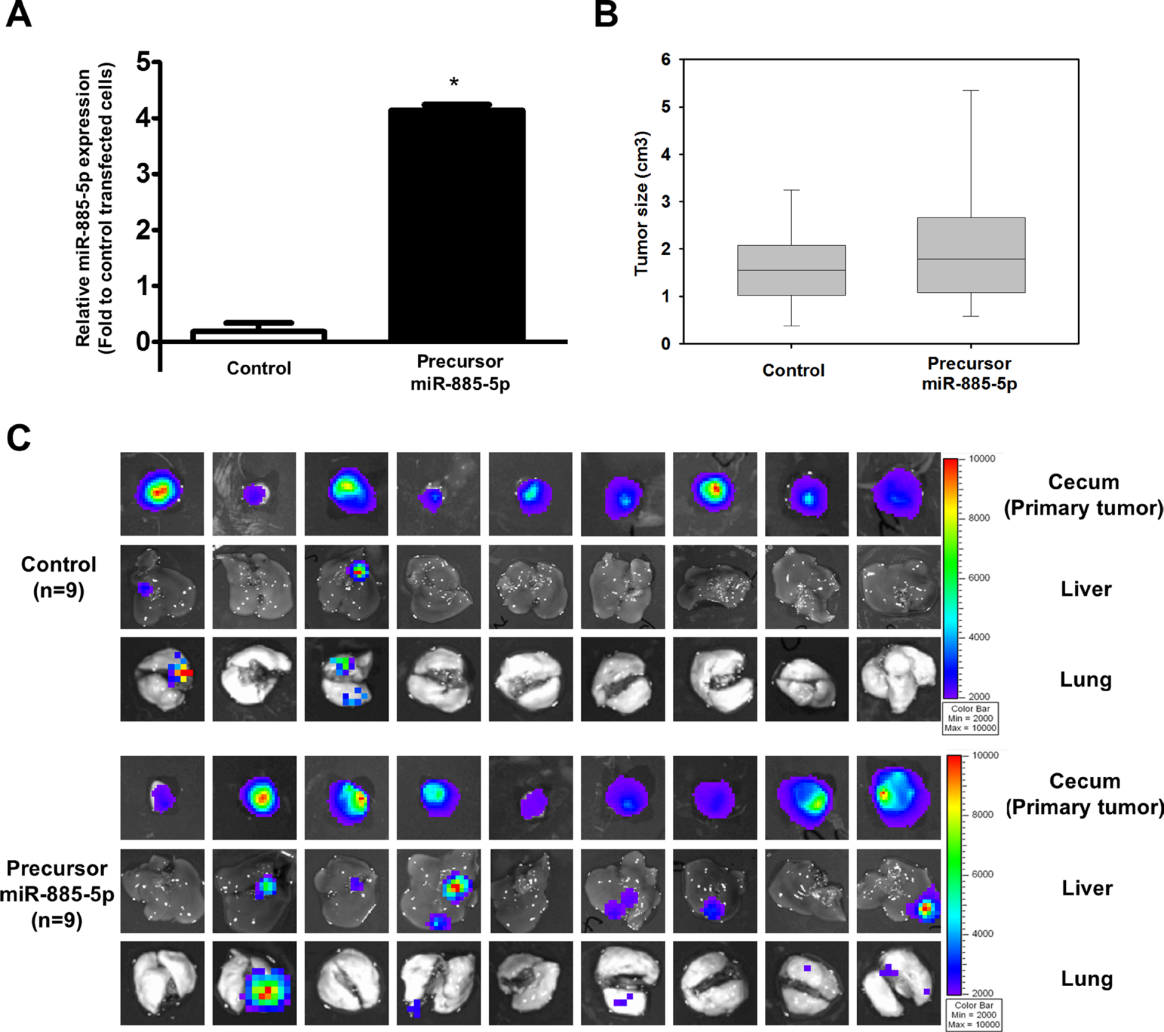
miR-885-5p induces CRC metastasis *in vivo* (**A**) Real-time RT-PCR was used to determine miR-885-5p expression level in luciferase-labelled HCT116 cell line stably transduced with empty lentivector (control) or precursor miR-885-5p. (**B**) The size of primary tumors developed from stable cells 6 weeks after orthotopically injected into the cecal wall of 9 mice per group. There was no significant difference in tumor size formed from control and miR-886-5p stable cells (*P* = 0.427). (**C**) The primary tumors, livers and lungs were excised and presence of tumor cells were detected by capturing the luciferase signal by the IVIS Imaging System. Primary tumors were detected in all mice injected with either control or precursor miR-885-5p stable cells. Liver and lung metastases were detected in only two mice of the control group, while 6 and 5 mice developed liver and lung metastases, respectively, in the miR-885-5p group.

To explore the role of miR-885-5p in tumor metastasis *in vivo*, luciferase-labelled HCT116 cell-line was transduced with empty lentivector (control) or precursor lenti-miR-885-5p (precursor miR-885-5p). 1 × 10^6^ cells of each stable clone were orthotopically injected into the cecal wall of 9 NOD-SCID mice and allowed to growth for 6 weeks. After 6 weeks, the mice were sacrificed and presence of tumor in cecum and metastatic tumor in liver and lung were detected by capturing the luciferase signal. We found that stable transfection of precursor miR-885-5p in HCT116 cells induced their metastatic capacity. Out of the 9 mice, 6 and 5 developed metastatic lesions in the liver and lung, respectively (Figure 6), whereas only 2 of control mice developed liver and lung metastases.

## DISCUSSION

Development of distant metastasis is the major cause of high mortality rate of CRC patients [32]. It is necessary to identify factors that confer metastatic capacity to tumor cells in order to understand the molecular mechanism leading to cancer metastasis, and to develop therapeutic agents to inhibit such transformation. Many miRNAs were identified as metastasis promoter or suppressor and thus provide us a new perspective on investigating the metastatic process [33, 34]. Therefore, this study aimed at identifying miRNAs responsible for CRC metastasis by comparing the miRNA profiles between primary CRCs and liver metastases.

Our miRNA microarray results showed that miR-885-5p was significantly overexpressed in liver metastasis when compared with primary CRC. This finding was also confirmed by quantitative PCR in another cohort of liver metastasis and primary CRC samples. Moreover, comparing the miR-885-5p level in two paired primary CRCs and liver metastases samples, both of them demonstrated much higher level in their liver metastases. These results clearly demonstrated that high miR-885-5p was associated with liver metastasis. During this study was in progress, other research groups also reported upregulation of miR-885-5p level in liver metastases when compared with the paired primary CRC [35, 36]. In Hur et al's study, they further demonstrated serum miR-885-5p expression was statistically significantly associated with lymph node metastasis, distant metastasis, TNM stage, liver metastasis, and lymphatic invasion. On the other hand, tissue expression of miR-885-5p expression was not associated with clinico-pathological factors. In another recent rectal carcinoid tumor study, miR-885-5p was the most up-regulated miRNA in the rectal carcinoid tumors with lymphovascular invasion compared with that in those without invasion, and high miR-885-5p expression was independently associated with lymphovascular invasion [37]. Since we and Vychytilova-Faltejskova et al's study did not perform such correlation analysis, it is not known whether similar findings will be observed in our patient cohorts. However, our study may provide an explanation to such finding. Based on our functional experiments that high miR-885-5p CRC cells possessed higher potential to migrate and invade, we hypothesize the CRC cells with higher miR-885-5p level evade from the primary site and enter circulation system and spread to distant organ(s), hence its level in serum but not tissue correlated with metastatic features in Hur et al's study. Moreover, similar to Hur et al and Vychytilova-Faltejskova et al's studies, our miRNA profiles also demonstrated that miR-885-5p and miR-122 were highly expressed in liver metastases when compared with the primary CRC samples (Supplementary Table 1). Since our downstream experiments suggested that miR-122 did not play a functional role in CRC metastasis (data not shown), hence this study focused on miR-885-5p.

Metastasis development is a multi-step complex process which is initiated by loss of cell-to-cell adhesion. During EMT, tumor cells lose cell-cell adherence and undergo a remarkable rearrangement of the cytoskeleton to facilitate cell motility and invasion into adjacent connective tissues [7, 9, 10, 38]. It is known that the loss of E-cadherin is essential in initiating EMT and cellular detachment, so that the cancer cells can metastasize to distant organs [10]. In the present study we found that up-regulation of miR-885-5p had a significant impact on EMT. Overexpression of miR-885-5p resulted in decreased E-cadherin expression level, together with elevated levels of N-cadherin; the expression of Vimentin, suggesting that the non-metastatic CRC cell lines were undergoing EMT process and became more invasive. Down-regulation of E-cadherin was accompanied with increased expression level of its transcription regulator Snail, which further demonstrated that miR-885-5p up-regulation enhances the metastatic properties of CRC through activation of the EMT pathway. Knockdown of miR-885-5p not only conversely resulted in enhanced E-cadherin expression level but also showed the decreased levels of Snail and Vimentin in metastatic CRC cell lines, while inhibiting the cell motility and invasiveness. Thus, miR-885-5p probably targets regulator of EMT to enhance motility and invasiveness in CRC cells.

The migration ability increased upon the overexpression of miR-885-5p in wound healing and migration chamber assay, which was further demonstrated by stress fiber formation assay. High expression level of miR-885-5p increased stress fiber formation and finally, enhanced the cell migration. The role of Rho small GTP binding proteins in the regulation of actin cytoskeleton arrangement and cell migration has been well published [39]. Actin filaments are necessary for the maintenance of cytoskeleton networks, which regulate cell shape and cell motility. Although, the mechanisms for regulating N-cadherin expression in metastasis remain unclear, Bhowmick et al. showed that GTPase RhoA signaling was necessary for N-cadherin induction by TGFβ1 [40]. Our study demonstrated the overexpression of miR-885-5p in CRC cells stimulated the RhoA expression and formation of stress fiber without affecting the actin expression level. Inhibition of miR-885-5p in metastatic CRC cells conversely inhibited the expression of Rho small GTP binding proteins. For the first time, this study provided evidence supporting the role of miR-885-5p in the regulation of cytoskeleton through a Rho-GTPase signaling pathway. Rho-GTPase activity is determined by the amount of the GTP-binding, which is regulated by three sets of proteins, guanine exchange factors (GEFs), GTPase-activating proteins (GAPs) and guanine nucleotide-dissociation inhibitors (GDIs). Thus, miR-885-5p may also target members of GEFs, GAPs, or GDIs to increase GTP-bound Rho in miR-885-5p overexpressing cells. Further study is essential to identify which Rho regulator is targeted by miR-885-5p that leads to Rho-GTPase activation.

More importantly, this study demonstrated miR-885-5p activated EMT through targeting CPEB2, which is a negative regulator of TWIST1 [26]. TWIST1 is one of the regulators which promote EMT pathway in many cancers [22, 23]. We demonstrated that miR-885-5p overexpression resulted in CPEB2 repression, which in turn induced TWIST1 expression in CRC cell line; on the other hand, anti-miR knockdown of miR-885-5p induced CPEB2 expression and repressed TWIST1 level. Furthermore, our data from luciferase reporter assays confirmed that miR-885-5p directly reguated the transcription of CPEB2 by functionally targeting two binding sites (CPEB2-1 and CPEB2-2) in CPEB2 UTR region.

Indeed, miR-885-5p is shown to be highly expressed in the liver and is associated with acute liver injury [41–43]. Similar to their findings, our results also demonstrated much higher miR-885-5p level in liver metastases and adjacent normal liver specimens than in CRC samples. We compared the miR-885-5p level in matched-liver metastases and non-tumor liver from 4 patients. The average relative miR-885-5p expressions in liver metastases and non-tumor liver were 0.0120 and 0.157, respectively. Furthermore, we detected the expression of paired HCC and non-tumor liver from 2 HCC patients (average: 0.769 and 0.332, respectively). We noticed the expression of miR-885-5p was much higher in adjacent normal liver when compared with the liver metastases samples, and even higher in paired-HCC and non-tumor liver specimens. Therefore, to prevent the influence of liver cells on miR-885-5p expression in liver-metastasis specimens, frozen samples were macrodissected with cryostat at −20°C to remove surrounding healthy liver tissue and three sections of 30 μm were collected for RNA extraction. Similar macrodissection procedure was also performed prior to RNA extraction in another recent colorectal cancer study which showed higher miR-885-5p expression in liver metastases when compared with the primary colorectal cancer specimens [36], confirming the higher expression of miR-885-5p in liver metastases was not due to tissue difference. In addition, miR-885-5p showed a higher expression in CRC cell-lines derived from metastatic site [SW620 (lymph node) and Colo205 (Ascites)], and our *in vitro* and *in vivo* experiment results which showed that miR-885-5p overexpression in CRC cell-lines induced migration, invasion and metastasis further indicated the high miR-885-5p level in liver metastases was due to the presence of miR-885-5p-high CRC cells which were more metastatic.

To conclude, we clearly showed in this study that miR-885-5p plays a crucial role in liver metastasis by enhancing cell motility, invasiveness, regulating EMT pathway through silencing its target gene CPEB2 which is a negative regulator of TWIST1. These findings, not only provide more information on molecular mechanism leading to CRC metastasis, but also potentiate the development of miR-885-5p inhibitor to treat advanced CRC.

## MATERIALS AND METHODS

### Patient samples and cell lines

Fresh randomized clinical tumor specimens were obtained from the Department of Surgery, Queen Mary Hospital, The University of Hong Kong between 2006 and 2009. All specimens were flash frozen in liquid nitrogen and stored at −80°C until use. The study was approved by the Institutional Review Board. Histological assessment was performed by two experienced pathologists. CCD-841-CoN, Colo 205, DLD-1, HCT 116, HCT-15, HT-29, SW480 and SW620 cell lines (American Type Culture Collection, ATCC) were cultured in medium according to ATCC.

### RNA extraction and real-time quantitative RT-PCR

Frozen samples were macrodissected with cryostat at −20°C to remove surrounding healthy tissue. Three sections of 30 μm were collected for RNA extraction. Total RNA that includes small RNAs from tissues and cells were isolated using mirVana™ miRNA Isolation Kit (Ambion, Austin, TX, USA) according to the manufacturer's instructions. All RNA samples were classified as good quality and were frozen at −80°C until further experiments if the 260/280 ratio higher than 2. In RT reactions, total miRNA was reverse transcribed into cDNA with Megaplex™ Primers Pools (Life Technologies Co.) for 754 unique miRNA. RNU6 snRNA and RNU48 were used on each experiment for endogenous control and an assay unrelated to mammalian species, ath-miR159a, providing a process control. Real-time qRT–PCR was performed using 7900HT (Life Technologies Co.). Experiments were performed in triplicate, independently. The relative expression of miR-885-5p was calculated by the 2−ΔCt method: 2^−(Ct of miR-885-5p – Ct of RNU6)^.

### Protein extraction and western blotting

Cells were lysed in RIPA buffer (Cell Signaling Technology, Danvers, MA) containing 1 mmol/l phenylmethylsulfonyl fluoride (PMSF) and 1X protease inhibitor cocktail (Cell Signaling Technology), the lysates were quantified using the BCA protein assay (Roche). Subcellular proteomes were isolated by compartment protein extraction kit (Millipore Co., Billerica, MA, USA). Primary antibodies used were against actin (Santa Cruz Biotechnology, Santa Cruz, USA), N-Cadherin, E-Cadherin, Snail, Vimentin, RhoA, RhoC, cdc42 and ROCK1 (Cell Signaling, Danvers, MA, USA).

### Wound-healing assay

Colorectal cell lines were seeded into 6-well plates at 1 × 10^5^ per well. Confluent monolayers were starved overnight and a single scratch wound was created by dragging a 1 ml plastic pipette tip across the well surface. Cells were washed with phosphate-buffered saline (PBS) twice and was assessed using an Olympus CKX41 microscope (Olympus, CenterValley, PA). Cell migration distance was measured using ImageJ software with different time points. The distance was calculated and expressed as a relative distance between times 0 and 48 hr.

### Transwells migration and matrigel invasion assays

Transwells with 8-μm pore size filters covered with (BD biosciences) or without matrigel (Corning Incorporated, Corning, NY, USA) were inserted into 24-well plates for invasion or migration assay, respectively. Medium (500 μl) containing 10% FBS as chemo-attractant was added to the lower chamber, and 300 μl of a serum-free cell suspension (5 × 10^4^ cells) was placed in the upper chamber. The plates were incubated at 37°C with 5% CO_2_ for 48h and cells that did not migrate or invade through the pores were removed by a cotton swab. Cells in the lower chamber were fixed in methanol and stained with 0.1% crystal violet. Number of invasive cells in five randomly selected fields from each chamber and were counted in each experiment. Experiments were performed independently at least three times.

### Immunofluorescence microscopy

Colorectal cells were fixed with 4% formaldehyde in PBS for 15 min and permeabilized with 0.1% Triton X-100 in PBS for 10 min. After blocking with 5% BSA for 1 h, the cells were incubated with anti-phalloidin Fluorescein Isothiocyanate Labelled (Sigma) overnight at 4°C. Coverslips were mounted with ProLong^®^ Gold anti-fade reagent (Life Technologies) containing nuclear stain 4′, 6-diamidino-2-phenylindole (DAPI) and then visualized using a Zeiss Axioplan 2 microscope (Carl Zeiss, Jena, Germany). Cells were scored as positive for intensive stress fibers when bundles of actin filaments were seen clearly emerging from the central portion of the cell. In all quantifications, only those cells presenting with free borders were considered, and at least 100 cells from randomly selected fields were evaluated.

### Luciferase labelled cell line

HCT 116 cells were transfected with firefly luciferase-expressing plasmid (Promega) and selected by puromycin. A single clone was picked out and grown in the medium containing 1 μg/ml puromycin.

### Lentiviral vectors and transduction

The pCDH-CMV-MCS-EF1-copGFP lentivector with CMV promoter to introduce short hairpin lenti-miR microRNA precursors (Pre-miR) and anti-microRNA (miZIP) were obtained from System Biosciences (SBI, Mountain View, CA, USA). The pseudoviral particle producer 293TN cell line (SBI) was transiently co-transfected with plasmid DNA and pPackH1 Packaging Plasmid Mix (SBI) using Lipofectamine™ 2000 (Life Technologies Co., Carlsbad, CA, USA). The virus-containing supernatants were collected and filtered 48 h after transfection and then used to infect colorectal cell lines in the presence of 5 μg/ml polybrene (Sigma, USA). After infection, the cells were sorted by MoFlo™ XDP (Beckman Coulter, Brea, CA, USA) by green fluorescent protein (GFP) signal in order to select out the stable gain-of-function cells.

### Luciferase reporter assay

miRNA-3′-UTR binding sites were identified by microRNA.org. pMIR-REPORT™ System (Life Technologies Co.) was used for reporter assay according to manufacturer instructions and co-transfected with *Renilla luciferase* vectors, phRL-TK (Promega, Madison, WI, USA), as an internal transfection control. Reporter plasmids were created by cloning putative miR-885-5p target sites in the CPEB2 3′-UTR into pMIR-REPORTER vector downstream of the firefly *luciferase* gene in pMIR-REPORTER vector. Westermann's lab identified miR-885-5p target sites in the CDK2 and MCM5 were used as positive control with modifications of restriction enzyme: SpeI-3′UTR target site-MluI [30].

Cells were plated in 24-well plates, and transfected with 200ng of either pMIR empty, pMIR-CPEB2, pMIR-CDK2 or pMIR-MCM5 using Lipofectamine^®^ 2000 (Life Technologies Co.), or miRNA mimics. After 24-h post transfection, cells were lysed in passive cell lysis buffer (Promega). Lysates were transferred into 96-well plates and processed using the Dual-Luciferase Reporter Assay System (Promega) according to the manufacturer instructions. The luminescence was measured in a FluorStar Optima Microplate Luminometer. Firefly luciferase activity was normalized to *Renilla luciferase* activity. Transfections were done in triplicate and repeated in three individual experiments.

### Animal experiments

Male 6- to 8-week-old NOD.CB17-Prkdcscid/J NOD/SCID mice (The Jackson Laboratory, Bar Harbor, Maine) were purchased from Laboratory Animal Unit. Colorectal HCT116 cells (1 × 10^6^ cells) were suspended in 50 μl of serum-free medium per 50 μl Matrigel (BD Biosciences, Sparks, MD) and injected orthotopically into the cecal wall of NOD/SCID mice to determine tumor metastasis. All animal experiments were approved by Committee on the Use of Live Animals in Teaching and Research. Mice were sacrificed 6 weeks after injection and the primary tumor, liver and lung bioluminescence signal were detected by Xenogen *in vivo* imaging system 100 series (Caliper Life Sciences, Inc, Hopkinton, MA).

### Statistical analysis

The results were performed in triplicate and were presented as mean ± SEM of at least three independent experiments. Different values among each group were analysed by using Student's *t*-test or one-way ANOVA. Continuous data were expressed as median, and Mann-Whitney U test was used to compare between groups. The χ^2^ test (or Fisher exact test where appropriate) was used for nominal variables. All statistical analyses were performed by SPSS 16 statistical software (SPSS, Chicago, IL). *P* < 0.05 was considered statistically significant.

## SUPPLEMENTARY MATERIALS TABLES





## Abbreviations

CRC: colorectal cancer
miRNA: microRNA, CPEB2, cytoplasmic polyadenylation element binding protein 2
EMT: epithelial-mesenchymal transition
