# Supplementary material for: Identification of microRNA 885-5p as a novel regulator of tumor metastasis by targeting CPEB2 in colorectal cancer
Source: Oncotarget. 2017 Mar 2;8(16):26858–70. doi: 10.18632/oncotarget.15844 (PMC5432302; doi:10.18632/oncotarget.15844)
Supplement: Supplementary file 1 [file oncotarget-08-26858-s001.pdf]

Identification of microRNA 885-5p as a novel regulator of tumor metastasis by targeting CPEB2 in colorectal cancer

Supplementary Materials

Supplementary Table 1: Aberrantly expressed miRNAs between liver metastasis (TL) and primary CRC specimens (TC) (*N* = 5). See Supplementary_Table_1
