# Supplementary material for: Identification of microRNA 885-5p as a novel regulator of tumor metastasis by targeting CPEB2 in colorectal cancer
Source: Oncotarget. 2017 Mar 2;8(16):26858–70. doi: 10.18632/oncotarget.15844 (PMC5432302; doi:10.18632/oncotarget.15844)
Supplement: Supplementary file 2 [file oncotarget-08-26858-s002.docx]

**Supplementary Table 1: Aberrantly expressed miRNAs between liver metastasis (TL) and primary CRC specimens (TC) (N=5).**

| # | Detector | 166TL | 169TL | 181TL | 198TL | 236TL | mean | 196TC | 248TC | 264TC | 266TC | 273TC | mean | ddCt | Fold (TL/TC) |
| --- | --- | --- | --- | --- | --- | --- | --- | --- | --- | --- | --- | --- | --- | --- | --- |
| 350 | hsa-miR-885-5p-4395407 | 11.12217 | 15.443883 | 15.236274 | 17.285978 | 15.166934 | 14.8510478 | 17.285978 | 20.388506 |  | 21.38014 |  | 19.68487467 | 4.833826867 | 28.51851325 |
| 62 | hsa-miR-122-4395356 | 9.139378 | 13.437767 | 12.219001 | 14.332158 | 13.172385 | 12.4601378 | 14.332158 | 19.41456 | 18.138494 |  |  | 17.29507067 | 4.834932867 | 28.54038451 |
| 180 | hsa-miR-361-5p-4373035 | 17.18538 | 16.438226 | 15.27857 | 20.3837 | 14.188926 | 16.6949604 | 20.3837 | 16.417925 | 20.08771 | 21.27668 | 18.116978 | 19.2565986 | 2.5616382 | 5.903776884 |
| 133 | hsa-miR-204-4373094 | 16.124913 | 16.448076 | 16.223758 | 15.335124 | 14.157857 | 15.6579456 | 15.335124 | 20.392546 | 17.089178 | 21.45064 | 16.14072 | 18.0816416 | 2.423696 | 5.365438193 |
| 8 | hsa-miR-9-4373285 | 18.165183 | 15.437139 | 14.22179 | 19.3351 | 12.185029 | 15.8688482 | 19.3351 | 19.451975 | 17.127725 | 17.30054 | 18.12772 | 18.268612 | 2.3997638 | 5.277167587 |
| 163 | hsa-miR-328-4373049 | 17.160636 | 16.452554 | 14.28925 | 21.31535 | 13.15212 | 16.473982 | 21.31535 | 16.41021 | 18.13106 | 21.284475 | 16.18077 | 18.664373 | 2.190391 | 4.564291713 |
| 149 | hsa-miR-296-5p-4373066 | 15.170192 | 17.45344 | 16.31699 | 20.341617 | 14.207036 | 16.697855 | 20.341617 | 17.40322 | 20.097345 | 20.13425 | 16.16365 | 18.8280164 | 2.1301614 | 4.377664524 |
| 122 | hsa-miR-196b-4395326 | 8.123065 | 8.44031 | 7.245208 | 12.297122 | 7.156245 | 8.65239 | 12.297122 | 9.38001 | 9.104877 | 12.288552 | 10.137745 | 10.6416612 | 1.9892712 | 3.970363784 |
| 173 | hsa-miR-339-5p-4395368 | 15.0754 | 13.445522 | 12.228605 | 17.278773 | 12.18122 | 14.041904 | 17.278773 | 15.412476 | 15.109958 | 16.258445 | 15.160275 | 15.8439854 | 1.8020814 | 3.48722971 |
| 330 | hsa-miR-652-4395463 | 16.1366 | 14.464063 | 12.252113 | 18.332202 | 13.186137 | 14.874223 | 18.332202 | 14.404172 | 17.128805 | 18.301244 | 15.140126 | 16.6613098 | 1.7870868 | 3.451173012 |
| 70 | hsa-miR-128-4395327 | 18.149737 | 16.43168 | 15.271465 | 21.316573 | 15.167636 | 17.2674182 | 21.316573 | 17.37346 | 19.12867 | 19.274366 | 18.123955 | 19.0434048 | 1.7759866 | 3.424721329 |
| 22 | hsa-miR-20b-4373263 | 14.133742 | 13.41653 | 13.213947 | 15.281964 | 12.143506 | 13.6379378 | 15.281964 | 15.39872 | 16.09602 | 15.28068 | 14.122553 | 15.2359874 | 1.5980496 | 3.027337664 |
| 9 | hsa-miR-10a-4373153 | 10.127778 | 11.42267 | 10.265702 | 16.31978 | 9.156447 | 11.4584754 | 16.31978 | 12.41715 | 13.115337 | 12.297044 | 11.103684 | 13.050599 | 1.5921236 | 3.014928106 |
| 327 | hsa-miR-636-4395199 | 18.141265 | 17.429147 | 15.233897 | 19.29865 | 17.179337 | 17.4564592 | 19.29865 | 19.391985 | 20.12124 | 20.281472 | 16.148335 | 19.0483364 | 1.5918772 | 3.014413226 |
| 52 | hsa-miR-99a-4373008 | 11.13723 | 11.498567 | 13.233073 | 15.335199 | 10.198108 | 12.2804354 | 15.335199 | 11.416327 | 14.139051 | 16.289153 | 12.159594 | 13.8678648 | 1.5874294 | 3.005134161 |
| 60 | hsa-miR-106b-4373155 | 11.138669 | 11.435739 | 9.208896 | 12.29283 | 9.161513 | 10.6475294 | 12.29283 | 12.37925 | 12.096786 | 12.265138 | 12.136532 | 12.2341072 | 1.5865778 | 3.003360802 |
| 21 | hsa-miR-20a-4373286 | 7.135295 | 8.428403 | 6.237088 | 9.323417 | 5.160064 | 7.2568534 | 9.323417 | 8.384738 | 10.118013 | 8.258248 | 8.13106 | 8.8430952 | 1.5862418 | 3.002661408 |
| 66 | hsa-miR-125b-4373148 | 12.156232 | 11.431262 | 13.243293 | 14.293418 | 9.130935 | 12.051028 | 14.293418 | 10.34711 | 14.115942 | 17.28613 | 12.133112 | 13.6351424 | 1.5841144 | 2.998236942 |
| 61 | hsa-miR-107-4373154 | 17.14987 | 17.475484 | 14.266362 | 18.29759 | 15.164285 | 16.4707182 | 18.29759 | 17.409598 | 18.1277 | 18.29304 | 18.14029 | 18.0536436 | 1.5829254 | 2.995766957 |
| 351 | hsa-miR-886-3p-4395305 | 11.156604 | 9.428308 | 7.262822 | 15.317537 | 9.148135 | 10.4626812 | 15.317537 | 10.416033 | 11.11306 | 14.273475 | 9.106097 | 12.0452404 | 1.5825592 | 2.995006637 |
| 54 | hsa-miR-100-4373160 | 12.154956 | 11.452413 | 13.268344 | 15.308877 | 10.16262 | 12.469442 | 15.308877 | 11.370276 | 15.112596 | 16.299321 | 12.150845 | 14.048383 | 1.578941 | 2.987504736 |
| 47 | hsa-miR-92a-4395169 | 12.150328 | 12.439414 | 11.250356 | 15.314162 | 9.169965 | 12.064845 | 15.314162 | 12.382805 | 14.080022 | 13.257869 | 13.134676 | 13.6339068 | 1.5690618 | 2.967116966 |
| 311 | hsa-miR-579-4395509 | 18.135848 | 18.431806 | 16.252578 | 20.31775 | 17.155927 | 18.0587818 | 20.31775 | 19.362036 | 20.091745 | 20.249395 | 18.11369 | 19.6269232 | 1.5681414 | 2.96522463 |
| 118 | hsa-miR-193a-5p-4395392 | 16.14321 | 14.44686 | 14.248748 | 18.393863 | 15.143996 | 15.6753354 | 18.393863 | 16.4004 | 18.182207 | 17.300294 | 15.164864 | 17.0883256 | 1.4129902 | 2.662885138 |
| 38 | hsa-miR-29b-4373288 | 14.127888 | 16.428037 | 13.249477 | 16.278466 | 12.164042 | 14.449582 | 16.278466 | 15.39378 | 15.118597 | 16.292987 | 16.140934 | 15.8449528 | 1.3953708 | 2.630561538 |
| 136 | hsa-miR-210-4373089 | 10.164887 | 9.436412 | 6.267036 | 13.295009 | 8.18217 | 9.4691028 | 13.295009 | 10.41225 | 11.141592 | 11.314193 | 8.154473 | 10.8635034 | 1.3944006 | 2.628793102 |
| 17 | hsa-miR-18a-4395533 | 14.13595 | 14.441095 | 12.22735 | 15.300485 | 13.171773 | 13.8553306 | 15.300485 | 14.391816 | 16.12843 | 15.274774 | 15.134846 | 15.2460702 | 1.3907396 | 2.622130703 |
| 39 | hsa-miR-29c-4395171 | 10.144059 | 12.448772 | 9.240804 | 12.31097 | 10.185807 | 10.8660824 | 12.31097 | 12.406993 | 12.122333 | 12.301665 | 12.137843 | 12.2559608 | 1.3898784 | 2.62056592 |
| 29 | hsa-miR-26a-4395166 | 9.138436 | 8.41418 | 8.273336 | 11.316026 | 6.150092 | 8.658414 | 11.316026 | 8.366664 | 10.111148 | 11.28881 | 9.15036 | 10.0466016 | 1.3881876 | 2.617496485 |
| 169 | hsa-miR-335-4373045 | 15.150786 | 14.410705 | 14.23267 | 16.33042 | 12.176675 | 14.4602512 | 16.33042 | 16.36035 | 15.110355 | 16.287973 | 15.14528 | 15.8468756 | 1.3866244 | 2.614661891 |
| 48 | hsa-miR-93-4373302 | 9.109315 | 9.42418 | 8.22498 | 11.290305 | 8.164723 | 9.2427006 | 11.290305 | 9.353932 | 11.104013 | 11.257774 | 10.111638 | 10.6235324 | 1.3808318 | 2.604184746 |
| 174 | hsa-miR-340-4395369 | 15.098126 | 15.42864 | 14.25015 | 18.31948 | 14.159833 | 15.4512458 | 18.31948 | 16.354148 | 17.099355 | 17.258788 | 15.126343 | 16.8316228 | 1.380377 | 2.603363924 |
| 142 | hsa-miR-218-4373081 | 13.14266 | 14.429344 | 13.237092 | 14.269344 | 12.15081 | 13.44585 | 14.269344 | 13.367909 | 16.112703 | 17.26798 | 13.104916 | 14.8245704 | 1.3787204 | 2.600376281 |
| 85 | hsa-miR-139-5p-4395400 | 12.143006 | 13.439996 | 13.2396 | 15.321637 | 14.203727 | 13.6695932 | 15.321637 | 14.391707 | 15.102731 | 16.283439 | 14.140707 | 15.0480442 | 1.378451 | 2.599890748 |
| 147 | hsa-miR-224-4395210 | 12.124905 | 11.43935 | 10.252015 | 16.318797 | 10.156812 | 12.0583758 | 16.318797 | 11.367444 | 12.087352 | 14.281865 | 13.127947 | 13.436681 | 1.3783052 | 2.599628014 |
| 239 | hsa-miR-495-4381078 | 17.11798 | 17.44268 | 15.27659 | 19.2966 | 16.188542 | 17.0644784 | 19.2966 | 16.38609 | 20.142465 | 19.25648 | 17.13178 | 18.442683 | 1.3782046 | 2.599446747 |
| 194 | hsa-miR-375-4373027 | 13.161694 | 11.42898 | 11.249097 | 14.306144 | 8.176751 | 11.6645332 | 14.306144 | 13.386822 | 13.109367 | 15.2788 | 9.12349 | 13.0409246 | 1.3763914 | 2.596181777 |
| 209 | hsa-miR-425-4380926 | 10.136296 | 10.466405 | 9.27001 | 12.305553 | 8.114598 | 10.0585724 | 12.305553 | 11.361569 | 11.107206 | 12.293279 | 10.103339 | 11.4341892 | 1.3756168 | 2.59478823 |
| 247 | hsa-miR-502-5p-4373227 | 18.14705 | 19.46685 | 15.26445 | 20.33799 | 17.20068 | 18.083404 | 20.33799 | 18.403224 | 20.147016 | 20.20983 | 18.16691 | 19.452994 | 1.36959 | 2.583971217 |
| 53 | hsa-miR-99b-4373007 | 14.209142 | 12.469139 | 13.231725 | 17.305455 | 11.139825 | 13.6710572 | 17.305455 | 12.372048 | 15.09292 | 16.235303 | 14.13717 | 15.0285792 | 1.357522 | 2.562446707 |
| 179 | hsa-miR-345-4395297 | 12.290355 | 11.427564 | 10.255334 | 14.301381 | 10.163297 | 11.6875862 | 14.301381 | 12.385718 | 13.095161 | 14.276782 | 11.101113 | 13.032031 | 1.3444448 | 2.539324555 |
| 59 | RNU44-4373384 | 8.12796 | 7.393922 | 6.22873 | 9.251617 | 7.108107 | 7.6220672 | 9.251617 | 9.375681 | 9.132785 | 8.27368 | 8.101025 | 8.8269576 | 1.2048904 | 2.305197564 |
| 123 | hsa-miR-197-4373102 | 13.112584 | 11.46967 | 11.206567 | 15.307353 | 11.191087 | 12.4574522 | 15.307353 | 12.391114 | 14.122543 | 14.325832 | 12.154349 | 13.6602382 | 1.202786 | 2.301837518 |
| 144 | hsa-miR-221-4373077 | 13.131812 | 12.443231 | 11.274398 | 16.34163 | 12.154132 | 13.0690406 | 16.34163 | 12.408612 | 14.128461 | 15.307643 | 13.155222 | 14.2683136 | 1.199273 | 2.296239302 |
| 6 | hsa-let-7g-4395393 | 10.116189 | 11.413644 | 10.24764 | 13.310382 | 9.141667 | 10.8459044 | 13.310382 | 11.389697 | 12.112756 | 12.268436 | 11.139119 | 12.044078 | 1.1981736 | 2.294490129 |
| 98 | hsa-miR-148b-4373129 | 17.1346 | 17.426774 | 16.281715 | 18.28193 | 14.133752 | 16.6517542 | 18.28193 | 16.383986 | 19.121736 | 19.300153 | 16.15703 | 17.848967 | 1.1972128 | 2.292962562 |
| 340 | hsa-miR-744-4395435 | 14.131425 | 12.414028 | 13.236856 | 16.29864 | 13.146022 | 13.8453942 | 16.29864 | 14.394373 | 15.09922 | 16.269895 | 13.150431 | 15.0425118 | 1.1971176 | 2.29281126 |
| 56 | hsa-miR-103-4373158 | 12.121556 | 12.435636 | 11.248956 | 15.313611 | 11.167817 | 12.4575152 | 15.313611 | 12.390444 | 14.124117 | 14.296994 | 12.127297 | 13.6504926 | 1.1929774 | 2.286240856 |
| 379 | hsa-miR-511-4373236 | 17.116526 | 17.419607 | 18.25487 | 20.29858 | 17.16706 | 18.0513286 | 20.29858 | 18.38595 | 19.10312 | 21.296754 | 17.130528 | 19.2429864 | 1.1916578 | 2.28415064 |
| 19 | hsa-miR-19a-4373099 | 8.170068 | 9.419569 | 7.238944 | 9.294864 | 7.180825 | 8.260854 | 9.294864 | 9.401684 | 10.11637 | 9.29509 | 9.146246 | 9.4508508 | 1.1899968 | 2.281522371 |
| 236 | hsa-miR-491-5p-4381053 | 15.118108 | 13.422126 | 14.218385 | 18.303485 | 13.136239 | 14.8396686 | 18.303485 | 15.35835 | 15.104038 | 17.257154 | 14.124386 | 16.0294826 | 1.189814 | 2.281233304 |
| 224 | hsa-miR-483-5p-4395449 | 15.09702 | 15.42407 | 16.244422 | 19.32797 | 17.180287 | 16.6547538 | 19.32797 | 14.398596 | 20.125814 | 19.223584 | 16.145817 | 17.8443562 | 1.1896024 | 2.28089874 |
| 363 | hsa-miR-212-4373087 | 17.138694 | 15.462625 | 14.259147 | 20.33196 | 15.176032 | 16.4736916 | 20.33196 | 15.400973 | 18.146803 | 18.274434 | 16.1614 | 17.663114 | 1.1894224 | 2.280614178 |
| 177 | hsa-miR-342-3p-4395371 | 9.15485 | 8.45341 | 9.259847 | 12.31604 | 8.137902 | 9.4644098 | 12.31604 | 9.413004 | 10.119978 | 12.284503 | 9.133694 | 10.6534438 | 1.189034 | 2.280000278 |
| 68 | hsa-miR-127-3p-4373147 | 14.120312 | 13.438167 | 14.26383 | 17.315137 | 13.166076 | 14.4607044 | 17.315137 | 13.391684 | 17.12338 | 16.259174 | 14.14423 | 15.646721 | 1.1860166 | 2.275236635 |
| 33 | hsa-miR-28-3p-4395557 | 11.104104 | 10.431037 | 9.227256 | 14.269466 | 10.168925 | 11.0401576 | 14.269466 | 11.378216 | 12.09398 | 13.256993 | 10.122832 | 12.2242974 | 1.1841398 | 2.272278707 |
| 92 | hsa-miR-145-4395389 | 9.151183 | 7.421691 | 9.239082 | 11.32271 | 7.202018 | 8.8673368 | 11.32271 | 8.416388 | 10.08922 | 12.28688 | 8.140788 | 10.0511972 | 1.1838604 | 2.271838688 |
| 16 | hsa-miR-17-4395419 | 5.117514 | 5.391168 | 5.238532 | 7.294454 | 4.153182 | 5.43897 | 7.294454 | 6.373507 | 7.080608 | 6.25383 | 6.109513 | 6.6223824 | 1.1834124 | 2.271133324 |
| 152 | hsa-miR-301a-4373064 | 13.134072 | 13.450609 | 12.23063 | 16.320594 | 12.182044 | 13.4635898 | 16.320594 | 13.402928 | 14.117248 | 15.264238 | 14.126477 | 14.646297 | 1.1827072 | 2.270023448 |
| 223 | hsa-miR-455-5p-4378098 | 14.133258 | 15.456882 | 13.257291 | 18.327132 | 14.176885 | 15.0702896 | 18.327132 | 14.405896 | 17.1237 | 16.258752 | 15.149453 | 16.2529866 | 1.182697 | 2.270007399 |
| 317 | hsa-miR-598-4395179 | 16.140788 | 17.416955 | 14.239562 | 19.32538 | 13.1768 | 16.059897 | 19.32538 | 14.357064 | 19.122724 | 18.293702 | 15.11382 | 17.242538 | 1.182641 | 2.269919288 |
| 110 | hsa-miR-185-4395382 | 15.162421 | 13.417669 | 12.222982 | 17.307962 | 14.172496 | 14.456706 | 17.307962 | 14.372101 | 15.095455 | 16.268352 | 15.15255 | 15.639284 | 1.182578 | 2.269820166 |
| 97 | hsa-miR-148a-4373130 | 12.114415 | 14.438297 | 10.243312 | 15.323508 | 10.191718 | 12.46225 | 15.323508 | 14.396567 | 13.103382 | 13.2746 | 12.118725 | 13.6433564 | 1.1811064 | 2.26750605 |
| 159 | hsa-miR-323-3p-4395338 | 17.164283 | 15.4333 | 15.244647 | 20.281787 | 16.17816 | 16.8604354 | 20.281787 | 15.36186 | 20.153303 | 18.27261 | 16.136723 | 18.0412566 | 1.1808212 | 2.267057841 |
| 161 | hsa-miR-324-5p-4373052 | 16.129903 | 15.41667 | 16.25017 | 18.29067 | 15.178038 | 16.2530902 | 18.29067 | 15.370976 | 17.086004 | 19.270353 | 17.142403 | 17.4320812 | 1.178991 | 2.264183679 |
| 40 | hsa-miR-30b-4373290 | 9.153107 | 10.460517 | 9.26179 | 12.333223 | 8.186353 | 9.878998 | 12.333223 | 10.395531 | 11.117 | 11.291806 | 10.146729 | 11.0568578 | 1.1778598 | 2.262409056 |
| 41 | hsa-miR-30c-4373060 | 9.15925 | 9.435564 | 8.23523 | 11.302903 | 8.188702 | 9.2643298 | 11.302903 | 10.38603 | 11.11844 | 10.26771 | 9.126723 | 10.4403612 | 1.1760314 | 2.259543607 |
| 28 | hsa-miR-25-4373071 | 14.12407 | 13.42564 | 13.251271 | 16.291455 | 12.171908 | 13.8528688 | 16.291455 | 13.367556 | 15.093784 | 16.256595 | 14.132397 | 15.0283574 | 1.1754886 | 2.258693636 |
| 204 | hsa-miR-410-4378093 | 17.118613 | 16.423288 | 17.256156 | 19.296306 | 17.15402 | 17.4496766 | 19.296306 | 16.364928 | 20.073434 | 20.278188 | 17.108086 | 18.6241884 | 1.1745118 | 2.257164868 |
| 2 | hsa-let-7c-4373167 | 16.15381 | 16.43669 | 18.24809 | 21.31427 | 14.159673 | 17.2625066 | 21.31427 | 13.366498 | 19.09134 | 21.263368 | 17.13798 | 18.4346912 | 1.1721846 | 2.253526788 |
| 20 | hsa-miR-19b-4373098 | 5.162093 | 6.46892 | 4.266044 | 7.335471 | 4.167346 | 5.4799748 | 7.335471 | 6.391491 | 7.111414 | 6.277526 | 6.136055 | 6.6503914 | 1.1704166 | 2.250766818 |
| 108 | hsa-miR-183-4395380 | 16.152066 | 15.444429 | 14.259078 | 18.31294 | 14.194552 | 15.672613 | 18.31294 | 15.380427 | 17.11554 | 18.26495 | 15.13162 | 16.8410954 | 1.1684824 | 2.24775127 |
| 100 | hsa-miR-150-4373127 | 8.105506 | 8.409832 | 10.235543 | 10.299436 | 10.169904 | 9.4440442 | 10.299436 | 10.373091 | 10.111082 | 10.27786 | 11.153002 | 10.4428942 | 0.99885 | 1.998406397 |
| 67 | hsa-miR-126-4395339 | 5.138167 | 5.432167 | 5.25867 | 7.319083 | 5.13746 | 5.6571094 | 7.319083 | 5.394066 | 7.13634 | 7.291915 | 6.137621 | 6.655805 | 0.9986956 | 1.998192535 |
| 4 | hsa-let-7e-4395517 | 11.126561 | 11.447448 | 10.217613 | 14.296518 | 9.139478 | 11.2455236 | 14.296518 | 10.420273 | 13.111757 | 13.253432 | 10.122038 | 12.2408036 | 0.99528 | 1.993467383 |
| 158 | hsa-miR-320-4395388 | 9.131892 | 8.414833 | 6.222905 | 11.300399 | 8.159898 | 8.6459854 | 11.300399 | 8.38972 | 10.120893 | 10.266984 | 8.127415 | 9.6410822 | 0.9950968 | 1.993214259 |
| 87 | hsa-miR-140-5p-4373374 | 11.079501 | 10.407444 | 10.218541 | 14.298196 | 9.125249 | 11.0257862 | 14.298196 | 10.35103 | 13.11159 | 12.244437 | 10.096836 | 12.0204178 | 0.9946316 | 1.992571647 |
| 372 | hsa-miR-376c-4395233 | 12.115979 | 12.445898 | 12.250394 | 14.32221 | 12.195535 | 12.6660032 | 14.32221 | 12.385773 | 15.14107 | 14.322942 | 12.130421 | 13.6604832 | 0.99448 | 1.992362276 |
| 160 | hsa-miR-324-3p-4395272 | 15.12095 | 14.425558 | 14.240363 | 16.330276 | 13.15954 | 14.6553374 | 16.330276 | 14.38247 | 16.113163 | 17.27909 | 14.140475 | 15.6490948 | 0.9937574 | 1.991364615 |
| 181 | hsa-miR-362-3p-4395228 | 19.13867 | 18.42302 | 15.211917 | 20.311897 | 17.17193 | 18.0514868 | 20.311897 | 18.380423 | 19.11095 | 20.29152 | 17.130368 | 19.0450316 | 0.9935448 | 1.991071183 |
| 116 | hsa-miR-192-4373108 | 7.11197 | 7.427722 | 4.235831 | 9.284368 | 5.153941 | 6.6427664 | 9.284368 | 7.376072 | 7.0987 | 7.281701 | 7.130434 | 7.634255 | 0.9914886 | 1.988235432 |
| 206 | hsa-miR-422a-4395408 | 13.139571 | 13.436775 | 11.237216 | 14.316816 | 12.152572 | 12.85659 | 14.316816 | 14.405072 | 14.105894 | 13.25949 | 13.143162 | 13.8460868 | 0.9894968 | 1.985492347 |
| 193 | hsa-miR-374b-4381045 | 11.12333 | 10.413171 | 9.216428 | 14.313365 | 9.156058 | 10.8444704 | 14.313365 | 11.391436 | 11.094846 | 12.252284 | 10.113005 | 11.8329872 | 0.9885168 | 1.984144091 |
| 30 | hsa-miR-26b-4395167 | 10.101426 | 11.446582 | 10.245836 | 14.31957 | 9.165309 | 11.0557446 | 14.31957 | 10.374167 | 12.098636 | 13.293605 | 10.133318 | 12.0438592 | 0.9881146 | 1.983591021 |
| 237 | hsa-miR-493-4395475 | 17.107837 | 16.420778 | 16.261718 | 20.293044 | 18.183064 | 17.6532882 | 20.293044 | 16.402724 | 20.098577 | 18.268872 | 18.131867 | 18.6390168 | 0.9857286 | 1.980313172 |
| 170 | hsa-miR-337-5p-4395267 | 18.165606 | 16.432733 | 16.22526 | 19.31695 | 17.17053 | 17.4622158 | 19.31695 | 16.374957 | 19.13298 | 19.27616 | 18.135308 | 18.447271 | 0.9850552 | 1.979389046 |
| 335 | hsa-miR-660-4380925 | 12.140385 | 12.43145 | 9.240333 | 14.29888 | 10.16393 | 11.6549956 | 14.29888 | 11.382392 | 12.103983 | 13.27238 | 12.13831 | 12.639189 | 0.9841934 | 1.978207003 |
| 231 | hsa-miR-487b-4378102 | 17.14601 | 17.45061 | 16.210048 | 19.316318 | 16.154527 | 17.2555026 | 19.316318 | 16.358035 | 19.10833 | 19.276784 | 17.131905 | 18.2382744 | 0.9827718 | 1.976258681 |
| 45 | hsa-miR-34a-4395168 | 13.117038 | 13.4457 | 11.2576 | 16.297366 | 12.171475 | 13.2578358 | 16.297366 | 13.375525 | 14.098924 | 15.279721 | 12.147335 | 14.2397742 | 0.9819384 | 1.975117388 |
| 107 | RNU48-4373383 | 6.168961 | 5.457645 | 5.248414 | 8.322871 | 4.181437 | 5.8758656 | 8.322871 | 6.400166 | 7.115333 | 6.293878 | 6.152281 | 6.8569058 | 0.9810402 | 1.973888092 |
| 50 | hsa-miR-96-4373372 | 19.146 | 18.45953 | 17.25323 | 20.292842 | 16.165395 | 18.2633994 | 20.292842 | 18.391554 | 19.124418 | 20.283555 | 18.129544 | 19.2443826 | 0.9809832 | 1.973810107 |
| 3 | hsa-let-7d-4395394 | 14.136533 | 13.433794 | 13.258489 | 17.30882 | 12.16415 | 14.0603572 | 17.30882 | 13.383385 | 15.09618 | 16.292361 | 13.1216 | 15.0404692 | 0.980112 | 1.972618542 |
| 73 | hsa-miR-130a-4373145 | 15.130585 | 14.447544 | 15.256984 | 17.339652 | 14.182406 | 15.2714342 | 17.339652 | 13.367144 | 17.12959 | 18.287255 | 15.13337 | 16.2514022 | 0.979968 | 1.972421659 |
| 34 | hsa-miR-28-5p-4373067 | 13.12369 | 12.407367 | 11.241079 | 16.2709 | 12.177777 | 13.0441626 | 16.2709 | 12.362313 | 14.097313 | 15.280297 | 12.109387 | 14.024042 | 0.9798794 | 1.97230053 |
| 210 | hsa-miR-429-4373203 | 10.126767 | 12.44371 | 8.242503 | 12.315126 | 8.167622 | 10.2591456 | 12.315126 | 11.394953 | 11.091991 | 11.27538 | 10.115956 | 11.2386812 | 0.9795356 | 1.971830579 |
| 37 | hsa-miR-29a-4395223 | 6.107207 | 7.40593 | 5.225686 | 8.273185 | 5.130257 | 6.428453 | 8.273185 | 7.36927 | 7.069532 | 7.21859 | 7.106068 | 7.407329 | 0.978876 | 1.970929265 |
| 14 | hsa-miR-15b-4373122 | 12.155959 | 12.46121 | 11.269696 | 15.313317 | 11.187701 | 12.4775766 | 15.313317 | 12.399067 | 13.13115 | 14.297061 | 12.135748 | 13.4552686 | 0.977692 | 1.969312414 |
| 99 | hsa-miR-149-4395366 | 14.122283 | 14.45695 | 13.263356 | 17.272634 | 14.201756 | 14.6633958 | 17.272634 | 14.411357 | 16.098756 | 17.276063 | 13.13937 | 15.639636 | 0.9762402 | 1.96733167 |
| 128 | hsa-miR-200a-4378069 | 10.123706 | 11.420549 | 7.220422 | 11.27481 | 7.140632 | 9.4360238 | 11.27481 | 10.36377 | 10.048622 | 10.25992 | 10.110415 | 10.4115074 | 0.9754836 | 1.966300202 |
| 1 | hsa-let-7a-4373169 | 13.272466 | 13.461626 | 12.245898 | 16.324645 | 11.160165 | 13.29296 | 16.324645 | 12.404824 | 15.15557 | 15.314231 | 12.13263 | 14.26638 | 0.97342 | 1.963489659 |
| 289 | hsa-miR-539-4378103 | 17.12174 | 15.407476 | 15.216903 | 20.310683 | 15.127162 | 16.6367928 | 20.310683 | 14.33865 | 18.047914 | 20.257096 | 15.09032 | 17.6089326 | 0.9721398 | 1.961748096 |
| 129 | hsa-miR-200b-4395362 | 8.13337 | 8.470756 | 5.254466 | 9.323129 | 5.17985 | 7.2723142 | 9.323129 | 8.378457 | 8.135673 | 8.243144 | 7.14119 | 8.2443186 | 0.9720044 | 1.96156399 |
| 287 | hsa-miR-532-3p-4395466 | 14.16023 | 13.4506 | 11.266654 | 16.30782 | 12.174695 | 13.4719998 | 16.30782 | 13.386248 | 14.109886 | 15.262803 | 13.152676 | 14.4438866 | 0.9718868 | 1.961404102 |
| 58 | hsa-miR-106a-4395280 | 5.130202 | 6.442567 | 5.254035 | 7.308978 | 4.150231 | 5.6572026 | 7.308978 | 6.37472 | 7.080059 | 6.260844 | 6.111163 | 6.6271528 | 0.9699502 | 1.95877298 |
| 18 | hsa-miR-18b-4395328 | 14.22199 | 15.432384 | 13.268005 | 15.372628 | 13.169013 | 14.292804 | 15.372628 | 15.386952 | 15.07651 | 15.282483 | 15.106026 | 15.2449198 | 0.9521158 | 1.934707945 |
| 79 | hsa-miR-135a-4373140 | 19.130797 | 18.41979 | 17.221256 | 22.30579 | 16.14072 | 18.6436706 | 22.30579 | 18.398493 | 19.114275 | 19.278997 | 18.14313 | 19.448137 | 0.8044664 | 1.746499706 |
| 211 | hsa-miR-431-4395173 | 15.176022 | 15.47494 | 13.248624 | 17.373183 | 14.212726 | 15.097099 | 17.373183 | 13.403643 | 17.16853 | 16.324765 | 15.219204 | 15.897865 | 0.800766 | 1.742025811 |
| 121 | hsa-miR-195-4373105 | 11.0973 | 11.440546 | 11.272317 | 13.313907 | 10.17473 | 11.45976 | 13.313907 | 11.3919 | 12.117204 | 13.32059 | 11.146248 | 12.2579698 | 0.7982098 | 1.738941983 |
| 55 | hsa-miR-101-4395364 | 16.152531 | 17.427198 | 16.21874 | 17.283372 | 13.152482 | 16.0468646 | 17.283372 | 16.3917 | 17.1021 | 16.273307 | 17.149495 | 16.8399948 | 0.7931302 | 1.73283009 |
| 176 | hsa-let-7b-4395446 | 11.184235 | 10.430852 | 10.218536 | 15.264163 | 9.21092 | 11.2617412 | 15.264163 | 8.438399 | 13.171655 | 14.247497 | 9.150448 | 12.0544324 | 0.7926912 | 1.732302884 |
| 75 | hsa-miR-132-4373143 | 12.146657 | 11.434148 | 11.259628 | 15.294526 | 11.164655 | 12.2599228 | 15.294526 | 11.397812 | 13.117395 | 14.29991 | 11.137385 | 13.0494056 | 0.7894828 | 1.728454708 |
| 205 | hsa-miR-411-4381013 | 15.139619 | 14.433969 | 14.226718 | 16.343614 | 14.170855 | 14.862955 | 16.343614 | 13.38962 | 17.117292 | 17.27949 | 14.129677 | 15.6519386 | 0.7889836 | 1.727856733 |
| 74 | hsa-miR-130b-4373144 | 15.155867 | 14.456306 | 12.207227 | 16.314843 | 14.177016 | 14.4622518 | 16.314843 | 14.390637 | 15.12848 | 16.29073 | 14.131052 | 15.2511484 | 0.7888966 | 1.72775254 |
| 315 | hsa-miR-590-5p-4395176 | 11.10918 | 11.427856 | 10.250152 | 12.274781 | 10.155637 | 11.0435212 | 12.274781 | 11.366751 | 12.12001 | 12.249808 | 11.146428 | 11.8315556 | 0.7880344 | 1.726720289 |
| 172 | hsa-miR-339-3p-4395295 | 13.15425 | 11.41633 | 11.231416 | 13.34075 | 11.151084 | 12.058766 | 13.34075 | 13.406842 | 12.078897 | 13.29016 | 12.110703 | 12.8454704 | 0.7867044 | 1.725129184 |
| 243 | hsa-miR-500-4395539 | 15.11964 | 15.434246 | 12.235696 | 17.278121 | 14.17092 | 14.8477246 | 17.278121 | 14.385275 | 15.104831 | 16.276124 | 15.127785 | 15.6344272 | 0.7867026 | 1.725127031 |
| 95 | hsa-miR-146b-5p-4373178 | 7.130849 | 6.425127 | 7.240949 | 11.332141 | 9.168837 | 8.2595806 | 11.332141 | 7.37966 | 9.106691 | 10.270719 | 7.133698 | 9.0445818 | 0.7850012 | 1.723093753 |
| 120 | hsa-miR-194-4373106 | 9.13034 | 9.439924 | 6.2293 | 11.2893 | 7.165557 | 8.6508842 | 11.2893 | 9.4113 | 9.081663 | 9.266872 | 8.123986 | 9.4346242 | 0.78374 | 1.721588087 |
| 114 | hsa-miR-190-4373110 | 19.150397 | 19.425798 | 17.252975 | 21.326762 | 16.161557 | 18.6634978 | 21.326762 | 18.389765 | 20.09447 | 19.27738 | 18.147217 | 19.4471188 | 0.783621 | 1.721446089 |
| 80 | hsa-miR-135b-4395372 | 11.126107 | 11.42995 | 9.23927 | 15.301517 | 9.150348 | 11.2494384 | 15.301517 | 11.379311 | 12.085575 | 11.278957 | 10.11608 | 12.032288 | 0.7828496 | 1.720525888 |
| 245 | hsa-miR-501-5p-4373226 | 16.127663 | 16.45981 | 13.264785 | 18.30961 | 15.173392 | 15.867052 | 18.30961 | 15.398114 | 17.138563 | 17.265813 | 15.129856 | 16.6483912 | 0.7813392 | 1.718725561 |
| 225 | hsa-miR-484-4381032 | 8.136376 | 8.438846 | 6.237743 | 9.300174 | 7.143515 | 7.8513308 | 9.300174 | 8.357823 | 8.098804 | 9.274594 | 8.131052 | 8.6324894 | 0.7811586 | 1.718510421 |
| 195 | hsa-miR-376a-4373026 | 15.150262 | 15.447103 | 14.26974 | 16.290227 | 15.167502 | 15.2649668 | 16.290227 | 14.383118 | 17.130956 | 17.293498 | 15.13106 | 16.0457718 | 0.780805 | 1.718089271 |
| 130 | hsa-miR-200c-4395411 | 6.160123 | 5.44086 | 3.23009 | 7.308618 | 3.199412 | 5.0678206 | 7.308618 | 6.392617 | 6.11173 | 5.272401 | 4.153765 | 5.8478262 | 0.7800056 | 1.717137538 |
| 106 | hsa-miR-182-4395445 | 15.133153 | 14.416204 | 13.234569 | 16.285266 | 14.171735 | 14.6481854 | 16.285266 | 14.389756 | 16.104465 | 16.229602 | 14.1193 | 15.4256778 | 0.7774924 | 1.714148858 |
| 51 | hsa-miR-98-4373009 | 19.175856 | 17.441404 | 16.26107 | 20.329314 | 15.181159 | 17.6777606 | 20.329314 | 17.413523 | 18.096815 | 19.29242 | 17.140084 | 18.4544312 | 0.7766706 | 1.713172709 |
| 219 | hsa-miR-452-4395440 | 15.159072 | 14.430421 | 12.272147 | 17.331124 | 13.155759 | 14.4697046 | 17.331124 | 14.37107 | 14.103595 | 15.27941 | 15.142754 | 15.2455906 | 0.775886 | 1.712241265 |
| 288 | hsa-miR-532-5p-4380928 | 11.151503 | 11.458467 | 8.249614 | 13.335517 | 10.169373 | 10.8728948 | 13.335517 | 10.36039 | 11.120674 | 12.277955 | 11.143347 | 11.6475766 | 0.7746818 | 1.710812674 |
| 65 | hsa-miR-125a-5p-4395309 | 13.142869 | 11.449445 | 12.24668 | 15.322063 | 11.173997 | 12.6670108 | 15.322063 | 11.386725 | 14.089199 | 14.273282 | 12.136479 | 13.4415496 | 0.7745388 | 1.710643106 |
| 238 | hsa-miR-494-4395476 | 14.157967 | 14.455535 | 12.276822 | 15.391027 | 14.183305 | 14.0929312 | 15.391027 | 15.391043 | 15.103426 | 15.29238 | 13.15643 | 14.8668612 | 0.77393 | 1.709921388 |
| 192 | hsa-miR-374a-4373028 | 11.13526 | 13.444097 | 10.254865 | 13.288669 | 10.163031 | 11.6571844 | 13.288669 | 12.370585 | 12.10222 | 12.253236 | 12.128998 | 12.4287416 | 0.7715572 | 1.707111392 |
| 153 | hsa-miR-301b-4395503 | 17.13383 | 16.44739 | 15.25467 | 17.296416 | 16.18287 | 16.4630352 | 17.296416 | 17.399504 | 16.092907 | 18.268815 | 17.112328 | 17.233994 | 0.7709588 | 1.706403465 |
| 43 | hsa-miR-32-4395220 | 16.143334 | 19.450353 | 16.273975 | 20.346294 | 16.181374 | 17.679066 | 20.346294 | 18.3751 | 17.083753 | 18.28952 | 18.12941 | 18.4448154 | 0.7657494 | 1.700252956 |
| 5 | hsa-let-7f-4373164 | 14.141562 | 14.462888 | 13.2498 | 16.302255 | 13.114337 | 14.2541684 | 16.302255 | 13.38705 | 15.120193 | 16.301463 | 13.890507 | 15.0002936 | 0.7461252 | 1.67728192 |
| 221 | hsa-miR-454-4395434 | 9.133101 | 8.400922 | 8.22091 | 10.290088 | 9.15031 | 9.0390662 | 10.290088 | 9.36483 | 9.098295 | 10.264793 | 9.12413 | 9.6284272 | 0.589361 | 1.50458019 |
| 15 | hsa-miR-16-4373121 | 5.113774 | 5.43011 | 4.22917 | 7.291547 | 5.16206 | 5.4453322 | 7.291547 | 5.377732 | 6.092805 | 6.269105 | 5.12923 | 6.0320838 | 0.5867516 | 1.501861318 |
| 104 | hsa-miR-181a-4373117 | 14.108638 | 13.452405 | 14.288993 | 17.320884 | 12.173817 | 14.2689474 | 17.320884 | 13.419835 | 15.06959 | 15.335988 | 13.13046 | 14.8553514 | 0.586404 | 1.501499506 |
| 229 | hsa-miR-486-5p-4378096 | 14.12445 | 11.447572 | 12.225238 | 16.31628 | 14.17519 | 13.657746 | 16.31628 | 14.40771 | 14.084205 | 14.285197 | 12.118933 | 14.242465 | 0.584719 | 1.499746849 |
| 91 | hsa-miR-143-4395360 | 11.149443 | 10.421707 | 10.211595 | 12.289363 | 9.182824 | 10.6509864 | 12.289363 | 9.374043 | 11.100546 | 13.276452 | 10.132004 | 11.2344816 | 0.5834952 | 1.498475193 |
| 308 | hsa-miR-574-3p-4395460 | 9.145793 | 8.439456 | 9.244613 | 12.310811 | 7.17708 | 9.2635506 | 12.310811 | 8.413498 | 10.117212 | 10.266803 | 8.123997 | 9.8464642 | 0.5829136 | 1.497871227 |
| 111 | hsa-miR-186-4395396 | 9.120744 | 8.432792 | 8.233424 | 10.311525 | 9.151208 | 9.0499386 | 10.311525 | 9.372656 | 9.082359 | 10.266664 | 9.121436 | 9.630928 | 0.5809894 | 1.495874768 |
| 13 | hsa-miR-15a-4373123 | 13.13743 | 14.456876 | 12.261556 | 16.304809 | 12.167638 | 13.6656618 | 16.304809 | 13.403546 | 14.101785 | 14.260707 | 13.157147 | 14.2455988 | 0.579937 | 1.494783973 |
| 115 | hsa-miR-191-4395410 | 5.137866 | 5.453626 | 4.255412 | 6.29178 | 4.187186 | 5.065174 | 6.29178 | 6.38933 | 5.103784 | 6.304666 | 4.133908 | 5.6446936 | 0.5795196 | 1.494351565 |
| 167 | hsa-miR-331-3p-4373046 | 10.146807 | 9.463155 | 9.231656 | 12.279903 | 8.150336 | 9.8543714 | 12.279903 | 9.38371 | 10.07905 | 11.296735 | 9.127802 | 10.43344 | 0.5790686 | 1.493884489 |
| 198 | hsa-miR-379-4373349 | 16.13042 | 16.45105 | 15.22206 | 18.29378 | 16.169457 | 16.4533534 | 18.29378 | 15.387072 | 18.09622 | 18.25662 | 15.12619 | 17.0319764 | 0.578623 | 1.49342315 |
| 324 | hsa-miR-627-4380967 | 19.13926 | 18.433153 | 16.226043 | 18.288665 | 19.174615 | 18.2523472 | 18.288665 | 19.398753 | 18.075185 | 19.25813 | 19.134087 | 18.830964 | 0.5786168 | 1.493416732 |
| 88 | hsa-miR-141-4373137 | 11.119181 | 12.434864 | 9.250463 | 12.298381 | 8.14834 | 10.6502458 | 12.298381 | 11.369729 | 11.079477 | 10.253717 | 11.133112 | 11.2268832 | 0.5766374 | 1.491369146 |
| 27 | hsa-miR-24-4373072 | 5.154052 | 5.460151 | 5.24861 | 7.319421 | 4.186204 | 5.4736876 | 7.319421 | 4.396298 | 6.105318 | 7.283546 | 5.141776 | 6.0492718 | 0.5755842 | 1.49028081 |
| 101 | hsa-miR-152-4395170 | 14.12572 | 13.433578 | 13.252448 | 16.285468 | 13.177174 | 14.0548776 | 16.285468 | 12.374661 | 15.094492 | 16.27338 | 13.117924 | 14.629185 | 0.5743074 | 1.48896248 |
| 76 | hsa-miR-133a-4395357 | 15.166837 | 12.454242 | 13.225566 | 15.294606 | 13.182622 | 13.8647746 | 15.294606 | 13.376277 | 13.111744 | 17.266786 | 13.135056 | 14.4368938 | 0.5721192 | 1.486705816 |
| 184 | hsa-miR-365-4373194 | 14.1618 | 13.422014 | 14.275855 | 17.309037 | 14.175893 | 14.6689198 | 17.309037 | 13.383149 | 15.10663 | 16.260202 | 14.13231 | 15.2382656 | 0.5693458 | 1.483850556 |
| 78 | hsa-miR-134-4373299 | 14.159478 | 12.467412 | 14.327735 | 17.379173 | 14.191106 | 14.5049808 | 17.379173 | 12.400097 | 16.071237 | 16.228418 | 13.15876 | 15.047537 | 0.5425562 | 1.456550983 |
| 126 | hsa-miR-199a-3p-4395415 | 11.142838 | 10.421566 | 11.247203 | 12.28342 | 8.141482 | 10.6473018 | 12.28342 | 8.418728 | 12.118166 | 13.314592 | 9.106005 | 11.0481822 | 0.4008804 | 1.320313382 |
| 233 | hsa-miR-489-4395469 | 15.113313 | 13.424142 | 12.225806 | 15.302331 | 14.151877 | 14.0434938 | 15.302331 | 16.379298 | 13.082212 | 13.257997 | 14.111758 | 14.4267192 | 0.3832254 | 1.30425449 |
| 31 | hsa-miR-27a-4373287 | 11.100148 | 11.406412 | 11.228269 | 13.290681 | 10.145457 | 11.4341934 | 13.290681 | 10.375372 | 12.082056 | 12.233695 | 11.102404 | 11.8168416 | 0.3826482 | 1.303732782 |
| 182 | hsa-miR-362-5p-4378092 | 14.182765 | 14.441251 | 11.252223 | 15.33327 | 13.174199 | 13.6767416 | 15.33327 | 13.429108 | 14.09767 | 14.285123 | 13.140551 | 14.0571444 | 0.3804028 | 1.30170524 |
| 86 | hsa-miR-140-3p-4395345 | 13.173474 | 12.464658 | 12.247556 | 14.306018 | 12.198983 | 12.8781378 | 14.306018 | 12.398284 | 13.14101 | 14.287519 | 12.157431 | 13.2580524 | 0.3799146 | 1.301264825 |
| 213 | hsa-miR-449a-4373207 | 21.205863 | 19.421396 | 18.21649 | 15.281425 | 16.163903 | 18.0578154 | 15.281425 | 20.400706 | 19.102724 | 19.26279 | 18.12134 | 18.433797 | 0.3759816 | 1.297722216 |
| 137 | hsa-miR-214-4395417 | 11.149934 | 10.451883 | 11.2598 | 12.2923 | 9.164801 | 10.8637436 | 12.2923 | 8.37481 | 12.115625 | 13.276927 | 10.13711 | 11.2393544 | 0.3756108 | 1.297388719 |
| 49 | hsa-miR-95-4373011 | 13.149129 | 14.428623 | 10.253116 | 14.31961 | 11.186765 | 12.6674486 | 14.31961 | 12.38663 | 14.104417 | 12.27046 | 12.1271 | 13.0416434 | 0.3741948 | 1.296115962 |
| 132 | hsa-miR-203-4373095 | 9.128833 | 11.452632 | 7.269933 | 12.304479 | 9.165892 | 9.8643538 | 12.304479 | 9.396967 | 11.074966 | 8.27552 | 10.138625 | 10.2381114 | 0.3737576 | 1.295723241 |
| 23 | hsa-miR-21-4373090 | 8.158068 | 8.443147 | 7.252046 | 11.321265 | 6.174468 | 8.2697988 | 11.321265 | 6.377663 | 9.105226 | 9.288938 | 7.123713 | 8.643361 | 0.3735622 | 1.295547759 |
| 90 | hsa-miR-142-5p-4395359 | 17.142905 | 19.489553 | 16.237518 | 20.328944 | 16.18288 | 17.87636 | 20.328944 | 17.388846 | 18.09767 | 18.254712 | 17.1679 | 18.2476144 | 0.3712544 | 1.293476999 |
| 165 | hsa-miR-330-3p-4373047 | 18.115676 | 15.399797 | 16.29099 | 19.300704 | 16.202527 | 17.0619388 | 19.300704 | 16.323454 | 17.09697 | 18.243005 | 16.163445 | 17.4255156 | 0.3635768 | 1.286611777 |
| 334 | hsa-miR-655-4381015 | 18.165637 | 19.455022 | 18.228744 | 21.34756 | 18.193684 | 19.0781294 | 21.34756 | 17.36118 | 20.11679 | 19.266957 | 19.009617 | 19.4204208 | 0.3422914 | 1.267768564 |
| 352 | hsa-miR-886-5p-4395304 | 11.143105 | 10.447402 | 9.241338 | 15.309192 | 11.154935 | 11.4591944 | 15.309192 | 9.396649 | 10.09313 | 14.299201 | 9.14605 | 11.6488444 | 0.18965 | 1.140486998 |
| 292 | hsa-miR-542-5p-4395351 | 17.123492 | 16.43388 | 17.257858 | 18.337756 | 17.182042 | 17.2670056 | 18.337756 | 16.396536 | 18.125475 | 18.291467 | 16.130005 | 17.4562478 | 0.1892422 | 1.140164668 |
| 248 | hsa-miR-503-4373228 | 18.130397 | 17.453283 | 15.272052 | 18.2847 | 17.155667 | 17.2592198 | 18.2847 | 15.384726 | 19.136346 | 18.27924 | 16.14945 | 17.4468924 | 0.1876726 | 1.138924884 |
| 325 | hsa-miR-628-5p-4395544 | 14.0792 | 14.413988 | 15.20055 | 16.287559 | 14.113433 | 14.818946 | 16.287559 | 14.345576 | 15.059108 | 16.238921 | 13.097636 | 15.00576 | 0.186814 | 1.138247271 |
| 32 | hsa-miR-27b-4373068 | 14.15088 | 14.44637 | 14.275062 | 17.38402 | 13.185418 | 14.68835 | 17.38402 | 13.413773 | 14.096962 | 15.304845 | 14.161564 | 14.8722328 | 0.1838828 | 1.135936981 |
| 218 | hsa-miR-451-4373360 | 15.15893 | 11.435375 | 13.253383 | 17.28551 | 14.175847 | 14.261809 | 17.28551 | 13.378262 | 14.09871 | 14.256053 | 13.149687 | 14.4336444 | 0.1718354 | 1.126490698 |
| 117 | hsa-miR-193a-3p-4395361 | 15.153703 | 16.422388 | 14.257444 | 16.271419 | 15.16486 | 15.4539628 | 16.271419 | 15.349295 | 15.119264 | 15.261735 | 16.125885 | 15.6255196 | 0.1715568 | 1.126273181 |
| 89 | hsa-miR-142-3p-4373136 | 11.150178 | 12.440277 | 11.253097 | 14.286145 | 10.165007 | 11.8589408 | 14.286145 | 11.358176 | 12.115608 | 12.260533 | 10.111025 | 12.0262974 | 0.1673566 | 1.122998966 |
| 12 | MammU6-4395470 | 0.01944 | -0.116864 | -0.005353 | -0.29174 | -0.208871 | -0.1206776 | -0.29174 | -0.11383 | -0.206018 | 0.299649 | 0.154986 | -0.0313906 | 0.089287 | 1.063844286 |
| 35 | MammU6-4395470 | -0.096379 | -0.01659 | 0.076549 | 0.306274 | 0.143975 | 0.0827658 | 0.306274 | 0.350438 | -0.020404 | -0.124768 | 0.063176 | 0.1149432 | 0.0321774 | 1.022554261 |
| 119 | hsa-miR-193b-4395478 | 10.130338 | 9.424207 | 10.265151 | 14.32489 | 11.164664 | 11.06185 | 14.32489 | 8.376996 | 10.102464 | 12.270257 | 10.129214 | 11.0407642 | -0.0210858 | 0.985490726 |
| 208 | hsa-miR-424-4373201 | 19.153308 | 18.438253 | 17.262595 | 19.299037 | 16.163167 | 18.063272 | 19.299037 | 16.395728 | 19.093343 | 18.278997 | 17.143303 | 18.0420816 | -0.0211904 | 0.985419277 |
| 145 | hsa-miR-222-4395387 | 8.122838 | 6.407947 | 5.212526 | 10.293 | 6.151116 | 7.2374854 | 10.293 | 5.35864 | 6.971296 | 8.255471 | 5.094427 | 7.1945668 | -0.0429186 | 0.970689237 |
| 11 | MammU6-4395470 | 0.161198 | 0.458806 | -0.324628 | -0.185975 | -0.105485 | 0.0007832 | -0.185975 | -0.140294 | 0.101304 | 0.179612 | -0.166065 | -0.0422836 | -0.0430668 | 0.970589528 |
| 36 | MammU6-4395470 | -0.084244 | -0.32537 | 0.253442 | 0.1714565 | 0.17038 | 0.0371329 | 0.1714565 | -0.096328 | 0.12511 | -0.354509 | -0.052108 | -0.0412757 | -0.0784086 | 0.947101794 |
| 146 | hsa-miR-223-4395406 | 6.096625 | 5.417026 | 4.222251 | 10.282403 | 7.171193 | 6.6378996 | 10.282403 | 6.392556 | 6.117344 | 6.27664 | 2.112729 | 6.2363344 | -0.4015652 | 0.757036518 |
| 323 | hsa-miR-625-4395542 | 17.126925 | 18.469686 | 15.28088 | 19.338866 | 16.18344 | 17.2799594 | 19.338866 | 16.407145 | 17.100008 | 16.283717 | 15.136666 | 16.8532804 | -0.426679 | 0.743972397 |
| 83 | hsa-miR-138-4395395 | 15.136592 | 19.432306 | 18.249675 | 15.28728 | 18.15928 | 17.2530266 | 15.28728 | 16.38427 | 15.100544 | 16.292754 | 17.141083 | 16.0411862 | -1.2118404 | 0.431717536 |
| 339 | hsa-miR-708-4395452 | 16.109519 | 14.401205 | 17.221515 | 20.292968 | 16.163678 | 16.837777 | 20.292968 | 11.361685 | 15.098545 | 16.249017 | 13.116473 | 15.2237376 | -1.6140394 | 0.326682392 |
| 93 | hsa-miR-146a-4373132 | 8.13796 | 8.441654 | 9.266507 | 10.313377 | 7.164359 | 8.6647714 | 10.313377 | 7.363364 | 7.098596 | 5.244191 | 5.152543 | 7.0344142 | -1.6303572 | 0.323008223 |
| 42 | hsa-miR-31-4395390 | 15.152253 | 13.44709 | 12.260196 | 17.312537 | 14.163108 | 14.4670368 | 17.312537 | 10.390498 | 15.131008 | 9.285167 | 8.125966 | 12.0490352 | -2.4180016 | 0.187115166 |
| 7 | hsa-miR-1-4395333 | 20.15816 | 19.43318 | #VALUE! | 21.32222 | 17.19164 | #VALUE! | 21.32222 | 18.40812 | 19.10092 | 22.225267 | 18.14814 | 19.8409334 | #VALUE! | #VALUE! |
| 10 | hsa-miR-10b-4395329 | 13.165772 | 15.480356 | 13.281917 | #VALUE! | 11.185859 | #VALUE! | #VALUE! | 13.432711 | 14.11045 | 13.322776 | 14.142632 | #VALUE! | #VALUE! | #VALUE! |
| 24 | hsa-miR-22-4373079 | 12.20391 | 11.478078 | 10.245515 | 14.289535 | 11.198295 | 11.8830666 | 14.289535 | 11.419436 | 13.227857 | 13.250482 | #VALUE! | #VALUE! | #VALUE! | #VALUE! |
| 25 | hsa-miR-23a-4373074 | 13.0765 | #VALUE! | 13.438302 | 16.284232 | 12.135375 | #VALUE! | 16.284232 | 12.345713 | 15.045734 | 15.240443 | #VALUE! | #VALUE! | #VALUE! | #VALUE! |
| 26 | hsa-miR-23b-4373073 | 14.173268 | #VALUE! | 17.28905 | 19.311046 | 13.18538 | #VALUE! | 19.311046 | 14.425166 | 16.171026 | 17.302777 | 16.144562 | 16.6709154 | #VALUE! | #VALUE! |
| 44 | hsa-miR-33b-4395196 | #VALUE! | #VALUE! | #VALUE! | #VALUE! | #VALUE! | #VALUE! | #VALUE! | #VALUE! | #VALUE! | 21.248174 | #VALUE! | #VALUE! | #VALUE! | #VALUE! |
| 46 | hsa-miR-34c-5p-4373036 | #VALUE! | #VALUE! | #VALUE! | #VALUE! | #VALUE! | #VALUE! | #VALUE! | #VALUE! | #VALUE! | #VALUE! | #VALUE! | #VALUE! | #VALUE! | #VALUE! |
| 57 | hsa-miR-105-4395278 | #VALUE! | 14.428194 | #VALUE! | 19.317977 | #VALUE! | #VALUE! | 19.317977 | #VALUE! | #VALUE! | #VALUE! | #VALUE! | #VALUE! | #VALUE! | #VALUE! |
| 63 | hsa-miR-124-4373295 | 14.122464 | #VALUE! | 13.28771 | 15.343236 | #VALUE! | #VALUE! | 15.343236 | #VALUE! | 15.096096 | 16.303086 | 14.1802 | #VALUE! | #VALUE! | #VALUE! |
| 64 | hsa-miR-125a-3p-4395310 | 20.13261 | 17.42544 | 18.30818 | 21.277866 | 18.141843 | 19.0571878 | 21.277866 | 19.386527 | 18.08828 | 20.290296 | #VALUE! | #VALUE! | #VALUE! | #VALUE! |
| 69 | hsa-miR-127-5p-4395340 | #VALUE! | #VALUE! | #VALUE! | #VALUE! | #VALUE! | #VALUE! | #VALUE! | #VALUE! | #VALUE! | #VALUE! | #VALUE! | #VALUE! | #VALUE! | #VALUE! |
| 71 | hsa-miR-129-3p-4373297 | #VALUE! | #VALUE! | #VALUE! | #VALUE! | #VALUE! | #VALUE! | #VALUE! | 19.40105 | 19.121275 | #VALUE! | #VALUE! | #VALUE! | #VALUE! | #VALUE! |
| 72 | hsa-miR-129-5p-4373171 | #VALUE! | #VALUE! | #VALUE! | #VALUE! | #VALUE! | #VALUE! | #VALUE! | #VALUE! | #VALUE! | #VALUE! | #VALUE! | #VALUE! | #VALUE! | #VALUE! |
| 77 | hsa-miR-133b-4395358 | 17.198363 | 16.421663 | #VALUE! | 19.39065 | 15.181073 | #VALUE! | 19.39065 | 16.403308 | 17.106718 | 19.400335 | 18.236794 | 18.107561 | #VALUE! | #VALUE! |
| 81 | hsa-miR-136-4373173 | 20.12398 | 20.451528 | #VALUE! | 22.29432 | #VALUE! | #VALUE! | 22.29432 | 19.38865 | 20.112196 | 21.20693 | #VALUE! | #VALUE! | #VALUE! | #VALUE! |
| 82 | hsa-miR-137-4373301 | #VALUE! | #VALUE! | #VALUE! | #VALUE! | #VALUE! | #VALUE! | #VALUE! | #VALUE! | #VALUE! | #VALUE! | #VALUE! | #VALUE! | #VALUE! | #VALUE! |
| 84 | hsa-miR-139-3p-4395424 | #VALUE! | #VALUE! | #VALUE! | #VALUE! | #VALUE! | #VALUE! | #VALUE! | 13.45524 | #VALUE! | #VALUE! | #VALUE! | #VALUE! | #VALUE! | #VALUE! |
| 94 | hsa-miR-146b-3p-4395472 | 19.297942 | 17.417737 | 18.18472 | #VALUE! | #VALUE! | #VALUE! | #VALUE! | 18.39542 | #VALUE! | #VALUE! | #VALUE! | #VALUE! | #VALUE! | #VALUE! |
| 96 | hsa-miR-147b-4395373 | 18.182032 | #VALUE! | 15.252116 | 20.33126 | 17.187554 | #VALUE! | 20.33126 | 18.430224 | 18.12348 | 18.20952 | #VALUE! | #VALUE! | #VALUE! | #VALUE! |
| 102 | hsa-miR-153-4373305 | #VALUE! | #VALUE! | #VALUE! | #VALUE! | 19.181455 | #VALUE! | #VALUE! | #VALUE! | #VALUE! | #VALUE! | #VALUE! | #VALUE! | #VALUE! | #VALUE! |
| 103 | hsa-miR-154-4373270 | #VALUE! | #VALUE! | #VALUE! | #VALUE! | #VALUE! | #VALUE! | #VALUE! | #VALUE! | #VALUE! | #VALUE! | #VALUE! | #VALUE! | #VALUE! | #VALUE! |
| 105 | hsa-miR-181c-4373115 | 18.179423 | 19.462404 | #VALUE! | 20.283504 | 15.204438 | #VALUE! | 20.283504 | 17.424986 | 20.137273 | 20.303224 | 18.154416 | 19.2606806 | #VALUE! | #VALUE! |
| 109 | hsa-miR-184-4373113 | 21.255057 | #VALUE! | 16.23585 | #VALUE! | 19.136388 | #VALUE! | #VALUE! | #VALUE! | 21.19283 | 22.293554 | 19.29603 | #VALUE! | #VALUE! | #VALUE! |
| 112 | hsa-miR-187-4373307 | 19.131747 | 19.450887 | 17.26139 | #VALUE! | #VALUE! | #VALUE! | #VALUE! | #VALUE! | #VALUE! | #VALUE! | #VALUE! | #VALUE! | #VALUE! | #VALUE! |
| 113 | hsa-miR-188-3p-4395217 | #VALUE! | #VALUE! | #VALUE! | #VALUE! | #VALUE! | #VALUE! | #VALUE! | #VALUE! | #VALUE! | #VALUE! | #VALUE! | #VALUE! | #VALUE! | #VALUE! |
| 124 | hsa-miR-198-4395384 | #VALUE! | #VALUE! | #VALUE! | #VALUE! | #VALUE! | #VALUE! | #VALUE! | 18.398245 | 19.110597 | #VALUE! | #VALUE! | #VALUE! | #VALUE! | #VALUE! |
| 125 | hsa-miR-199a-5p-4373272 | 19.12925 | 17.434377 | #VALUE! | 19.332023 | 16.181508 | #VALUE! | 19.332023 | 16.398024 | #VALUE! | 21.229943 | 17.14583 | #VALUE! | #VALUE! | #VALUE! |
| 127 | hsa-miR-199b-5p-4373100 | #VALUE! | 19.43736 | #VALUE! | 21.346152 | 19.219704 | #VALUE! | 21.346152 | 15.439692 | 20.20897 | #VALUE! | 18.188954 | #VALUE! | #VALUE! | #VALUE! |
| 131 | hsa-miR-202-4395474 | #VALUE! | 18.470697 | 16.271007 | #VALUE! | 18.167718 | #VALUE! | #VALUE! | #VALUE! | #VALUE! | 21.249784 | #VALUE! | #VALUE! | #VALUE! | #VALUE! |
| 134 | hsa-miR-205-4373093 | #VALUE! | #VALUE! | #VALUE! | 19.334106 | #VALUE! | #VALUE! | 19.334106 | #VALUE! | #VALUE! | #VALUE! | #VALUE! | #VALUE! | #VALUE! | #VALUE! |
| 135 | hsa-miR-208b-4395401 | #VALUE! | #VALUE! | #VALUE! | #VALUE! | #VALUE! | #VALUE! | #VALUE! | #VALUE! | #VALUE! | #VALUE! | #VALUE! | #VALUE! | #VALUE! | #VALUE! |
| 138 | hsa-miR-215-4373084 | #VALUE! | #VALUE! | 7.184234 | #VALUE! | 10.145272 | #VALUE! | #VALUE! | 10.406802 | 10.18829 | 9.226638 | 8.186041 | #VALUE! | #VALUE! | #VALUE! |
| 139 | hsa-miR-216a-4395331 | 19.114432 | #VALUE! | #VALUE! | #VALUE! | 19.183797 | #VALUE! | #VALUE! | #VALUE! | 20.07993 | #VALUE! | #VALUE! | #VALUE! | #VALUE! | #VALUE! |
| 140 | hsa-miR-216b-4395437 | 20.066985 | #VALUE! | #VALUE! | 21.379886 | #VALUE! | #VALUE! | 21.379886 | #VALUE! | #VALUE! | #VALUE! | 18.17245 | #VALUE! | #VALUE! | #VALUE! |
| 141 | hsa-miR-217-4395448 | 21.184302 | #VALUE! | #VALUE! | #VALUE! | #VALUE! | #VALUE! | #VALUE! | #VALUE! | #VALUE! | #VALUE! | #VALUE! | #VALUE! | #VALUE! | #VALUE! |
| 143 | hsa-miR-219-5p-4373080 | #VALUE! | #VALUE! | #VALUE! | #VALUE! | #VALUE! | #VALUE! | #VALUE! | #VALUE! | #VALUE! | #VALUE! | #VALUE! | #VALUE! | #VALUE! | #VALUE! |
| 148 | hsa-miR-296-3p-4395212 | 18.11411 | #VALUE! | #VALUE! | #VALUE! | 18.191316 | #VALUE! | #VALUE! | #VALUE! | #VALUE! | #VALUE! | #VALUE! | #VALUE! | #VALUE! | #VALUE! |
| 150 | hsa-miR-299-3p-4373189 | #VALUE! | #VALUE! | #VALUE! | #VALUE! | #VALUE! | #VALUE! | #VALUE! | #VALUE! | #VALUE! | #VALUE! | #VALUE! | #VALUE! | #VALUE! | #VALUE! |
| 151 | hsa-miR-299-5p-4373188 | 20.14931 | 19.42815 | 18.258113 | #VALUE! | #VALUE! | #VALUE! | #VALUE! | 18.376444 | #VALUE! | 22.212068 | 17.138733 | #VALUE! | #VALUE! | #VALUE! |
| 154 | hsa-miR-302a-4378070 | #VALUE! | #VALUE! | #VALUE! | #VALUE! | #VALUE! | #VALUE! | #VALUE! | #VALUE! | #VALUE! | #VALUE! | #VALUE! | #VALUE! | #VALUE! | #VALUE! |
| 155 | ath-miR159a-4373390 | #VALUE! | #VALUE! | #VALUE! | #VALUE! | #VALUE! | #VALUE! | #VALUE! | #VALUE! | #VALUE! | #VALUE! | #VALUE! | #VALUE! | #VALUE! | #VALUE! |
| 156 | hsa-miR-302b-4378071 | #VALUE! | 19.418943 | #VALUE! | #VALUE! | 19.128953 | #VALUE! | #VALUE! | #VALUE! | #VALUE! | #VALUE! | #VALUE! | #VALUE! | #VALUE! | #VALUE! |
| 157 | hsa-miR-302c-4378072 | #VALUE! | #VALUE! | #VALUE! | #VALUE! | #VALUE! | #VALUE! | #VALUE! | #VALUE! | #VALUE! | 22.10909 | 19.198952 | #VALUE! | #VALUE! | #VALUE! |
| 162 | hsa-miR-326-4373050 | #VALUE! | #VALUE! | #VALUE! | #VALUE! | #VALUE! | #VALUE! | #VALUE! | #VALUE! | #VALUE! | #VALUE! | #VALUE! | #VALUE! | #VALUE! | #VALUE! |
| 164 | hsa-miR-329-4373191 | #VALUE! | #VALUE! | #VALUE! | #VALUE! | #VALUE! | #VALUE! | #VALUE! | #VALUE! | #VALUE! | #VALUE! | #VALUE! | #VALUE! | #VALUE! | #VALUE! |
| 166 | hsa-miR-330-5p-4395341 | #VALUE! | #VALUE! | #VALUE! | #VALUE! | #VALUE! | #VALUE! | #VALUE! | #VALUE! | #VALUE! | #VALUE! | #VALUE! | #VALUE! | #VALUE! | #VALUE! |
| 168 | hsa-miR-331-5p-4395344 | 16.15489 | 16.460973 | 14.250213 | 17.331569 | 16.245698 | 16.0886686 | 17.331569 | 16.433184 | 16.13645 | 16.300681 | #VALUE! | #VALUE! | #VALUE! | #VALUE! |
| 171 | hsa-miR-338-3p-4395363 | 20.159716 | #VALUE! | 17.212363 | #VALUE! | 16.15047 | #VALUE! | #VALUE! | 17.406462 | 20.127073 | 19.289506 | 17.137997 | #VALUE! | #VALUE! | #VALUE! |
| 175 | has-miR-155-4395459 | #VALUE! | 18.450864 | #VALUE! | #VALUE! | #VALUE! | #VALUE! | #VALUE! | 19.492964 | #VALUE! | #VALUE! | #VALUE! | #VALUE! | #VALUE! | #VALUE! |
| 178 | hsa-miR-342-5p-4395258 | 18.18803 | #VALUE! | #VALUE! | #VALUE! | 19.2591 | #VALUE! | #VALUE! | 19.427004 | 20.191324 | #VALUE! | #VALUE! | #VALUE! | #VALUE! | #VALUE! |
| 183 | hsa-miR-363-4378090 | #VALUE! | 19.42632 | #VALUE! | #VALUE! | #VALUE! | #VALUE! | #VALUE! | 19.395773 | #VALUE! | #VALUE! | #VALUE! | #VALUE! | #VALUE! | #VALUE! |
| 185 | hsa-miR-367-4373034 | #VALUE! | #VALUE! | #VALUE! | #VALUE! | #VALUE! | #VALUE! | #VALUE! | #VALUE! | #VALUE! | #VALUE! | #VALUE! | #VALUE! | #VALUE! | #VALUE! |
| 186 | hsa-miR-369-3p-4373032 | #VALUE! | #VALUE! | #VALUE! | 22.436237 | 20.161286 | #VALUE! | 22.436237 | #VALUE! | #VALUE! | #VALUE! | #VALUE! | #VALUE! | #VALUE! | #VALUE! |
| 187 | hsa-miR-369-5p-4373195 | #VALUE! | #VALUE! | #VALUE! | #VALUE! | #VALUE! | #VALUE! | #VALUE! | #VALUE! | #VALUE! | #VALUE! | #VALUE! | #VALUE! | #VALUE! | #VALUE! |
| 188 | hsa-miR-370-4395386 | #VALUE! | 14.503417 | #VALUE! | #VALUE! | #VALUE! | #VALUE! | #VALUE! | 14.411683 | 18.158136 | #VALUE! | #VALUE! | #VALUE! | #VALUE! | #VALUE! |
| 189 | hsa-miR-371-3p-4395235 | #VALUE! | #VALUE! | #VALUE! | #VALUE! | #VALUE! | #VALUE! | #VALUE! | #VALUE! | #VALUE! | #VALUE! | #VALUE! | #VALUE! | #VALUE! | #VALUE! |
| 190 | hsa-miR-372-4373029 | #VALUE! | 17.430456 | 19.22263 | #VALUE! | #VALUE! | #VALUE! | #VALUE! | #VALUE! | 20.131616 | 19.23775 | #VALUE! | #VALUE! | #VALUE! | #VALUE! |
| 191 | hsa-miR-373-4378073 | #VALUE! | #VALUE! | #VALUE! | #VALUE! | #VALUE! | #VALUE! | #VALUE! | #VALUE! | #VALUE! | #VALUE! | #VALUE! | #VALUE! | #VALUE! | #VALUE! |
| 196 | hsa-miR-376b-4373196 | 19.16353 | #VALUE! | #VALUE! | #VALUE! | #VALUE! | #VALUE! | #VALUE! | #VALUE! | #VALUE! | #VALUE! | #VALUE! | #VALUE! | #VALUE! | #VALUE! |
| 197 | hsa-miR-377-4373025 | #VALUE! | #VALUE! | #VALUE! | #VALUE! | #VALUE! | #VALUE! | #VALUE! | 19.37459 | #VALUE! | #VALUE! | #VALUE! | #VALUE! | #VALUE! | #VALUE! |
| 199 | hsa-miR-380-4373022 | #VALUE! | #VALUE! | #VALUE! | #VALUE! | #VALUE! | #VALUE! | #VALUE! | #VALUE! | #VALUE! | #VALUE! | #VALUE! | #VALUE! | #VALUE! | #VALUE! |
| 200 | hsa-miR-381-4373020 | #VALUE! | #VALUE! | 18.274 | #VALUE! | #VALUE! | #VALUE! | #VALUE! | 19.384764 | #VALUE! | #VALUE! | 18.20105 | #VALUE! | #VALUE! | #VALUE! |
| 201 | hsa-miR-382-4373019 | #VALUE! | 15.46034 | #VALUE! | #VALUE! | 17.197198 | #VALUE! | #VALUE! | 15.419783 | #VALUE! | #VALUE! | 18.15351 | #VALUE! | #VALUE! | #VALUE! |
| 202 | hsa-miR-383-4373018 | 20.172572 | 19.505815 | #VALUE! | #VALUE! | 17.170827 | #VALUE! | #VALUE! | #VALUE! | #VALUE! | #VALUE! | #VALUE! | #VALUE! | #VALUE! | #VALUE! |
| 203 | hsa-miR-409-5p-4395442 | #VALUE! | #VALUE! | #VALUE! | #VALUE! | 17.165933 | #VALUE! | #VALUE! | #VALUE! | #VALUE! | #VALUE! | #VALUE! | #VALUE! | #VALUE! | #VALUE! |
| 207 | hsa-miR-423-5p-4395451 | 15.334372 | 15.485784 | 12.253526 | #VALUE! | 14.152839 | #VALUE! | #VALUE! | 15.342454 | 17.140405 | #VALUE! | 14.154122 | #VALUE! | #VALUE! | #VALUE! |
| 212 | hsa-miR-433-4373205 | 16.141894 | 16.461877 | 14.242564 | #VALUE! | 16.17471 | #VALUE! | #VALUE! | 15.362536 | 18.113115 | 18.35492 | #VALUE! | #VALUE! | #VALUE! | #VALUE! |
| 214 | hsa-miR-449b-4381011 | 20.207705 | 19.49201 | #VALUE! | 18.31408 | 18.168748 | #VALUE! | 18.31408 | 19.46649 | 20.103284 | 20.308377 | 18.15435 | 19.2693162 | #VALUE! | #VALUE! |
| 215 | hsa-miR-450a-4395414 | #VALUE! | #VALUE! | #VALUE! | #VALUE! | 18.17967 | #VALUE! | #VALUE! | 18.380137 | 20.092447 | 21.31729 | #VALUE! | #VALUE! | #VALUE! | #VALUE! |
| 216 | hsa-miR-450b-3p-4395319 | #VALUE! | #VALUE! | #VALUE! | #VALUE! | #VALUE! | #VALUE! | #VALUE! | #VALUE! | #VALUE! | #VALUE! | #VALUE! | #VALUE! | #VALUE! | #VALUE! |
| 217 | hsa-miR-450b-5p-4395318 | #VALUE! | 19.491937 | 18.258624 | 21.248107 | 19.268647 | #VALUE! | 21.248107 | 19.399306 | #VALUE! | 21.28644 | 18.108536 | #VALUE! | #VALUE! | #VALUE! |
| 220 | hsa-miR-453-4395429 | #VALUE! | #VALUE! | #VALUE! | #VALUE! | #VALUE! | #VALUE! | #VALUE! | #VALUE! | #VALUE! | #VALUE! | #VALUE! | #VALUE! | #VALUE! | #VALUE! |
| 222 | hsa-miR-455-3p-4395355 | 16.12886 | 16.444677 | 14.213762 | #VALUE! | 15.186316 | #VALUE! | #VALUE! | 15.379187 | #VALUE! | 18.199205 | 16.11192 | #VALUE! | #VALUE! | #VALUE! |
| 226 | hsa-miR-485-3p-4378095 | 20.175708 | 17.403184 | 17.252166 | #VALUE! | #VALUE! | #VALUE! | #VALUE! | 18.379683 | #VALUE! | 21.325658 | 17.12664 | #VALUE! | #VALUE! | #VALUE! |
| 227 | hsa-miR-485-5p-4373212 | #VALUE! | #VALUE! | #VALUE! | #VALUE! | #VALUE! | #VALUE! | #VALUE! | #VALUE! | #VALUE! | #VALUE! | #VALUE! | #VALUE! | #VALUE! | #VALUE! |
| 228 | hsa-miR-486-3p-4395204 | 17.157714 | 14.441457 | #VALUE! | 19.31384 | 16.197465 | #VALUE! | 19.31384 | 18.434588 | 18.086153 | 18.29098 | 15.15164 | 17.8554402 | #VALUE! | #VALUE! |
| 230 | hsa-miR-487a-4378097 | #VALUE! | 15.439073 | #VALUE! | #VALUE! | 14.099864 | #VALUE! | #VALUE! | 17.402056 | #VALUE! | #VALUE! | #VALUE! | #VALUE! | #VALUE! | #VALUE! |
| 232 | hsa-miR-488-4395468 | #VALUE! | 19.41503 | #VALUE! | #VALUE! | #VALUE! | #VALUE! | #VALUE! | #VALUE! | #VALUE! | #VALUE! | #VALUE! | #VALUE! | #VALUE! | #VALUE! |
| 234 | hsa-miR-490-3p-4373215 | 18.17636 | #VALUE! | #VALUE! | #VALUE! | #VALUE! | #VALUE! | #VALUE! | #VALUE! | #VALUE! | #VALUE! | #VALUE! | #VALUE! | #VALUE! | #VALUE! |
| 235 | hsa-miR-491-3p-4395471 | #VALUE! | #VALUE! | #VALUE! | #VALUE! | #VALUE! | #VALUE! | #VALUE! | #VALUE! | #VALUE! | #VALUE! | #VALUE! | #VALUE! | #VALUE! | #VALUE! |
| 240 | hsa-miR-496-4386771 | #VALUE! | #VALUE! | #VALUE! | #VALUE! | #VALUE! | #VALUE! | #VALUE! | #VALUE! | #VALUE! | #VALUE! | #VALUE! | #VALUE! | #VALUE! | #VALUE! |
| 241 | hsa-miR-499-3p-4395538 | #VALUE! | #VALUE! | #VALUE! | #VALUE! | #VALUE! | #VALUE! | #VALUE! | #VALUE! | #VALUE! | #VALUE! | #VALUE! | #VALUE! | #VALUE! | #VALUE! |
| 242 | hsa-miR-499-5p-4381047 | #VALUE! | #VALUE! | #VALUE! | 17.30853 | 17.175332 | #VALUE! | 17.30853 | #VALUE! | #VALUE! | #VALUE! | #VALUE! | #VALUE! | #VALUE! | #VALUE! |
| 244 | hsa-miR-501-3p-4395546 | #VALUE! | #VALUE! | #VALUE! | #VALUE! | #VALUE! | #VALUE! | #VALUE! | #VALUE! | #VALUE! | #VALUE! | #VALUE! | #VALUE! | #VALUE! | #VALUE! |
| 246 | hsa-miR-502-3p-4395194 | 19.136298 | 17.434553 | 15.2555 | 21.336917 | 16.164254 | 17.8655044 | 21.336917 | 17.395117 | 18.125433 | 19.274385 | #VALUE! | #VALUE! | #VALUE! | #VALUE! |
| 249 | hsa-miR-504-4395195 | #VALUE! | #VALUE! | #VALUE! | #VALUE! | 16.092328 | #VALUE! | #VALUE! | #VALUE! | #VALUE! | #VALUE! | #VALUE! | #VALUE! | #VALUE! | #VALUE! |
| 250 | hsa-miR-505-4395200 | #VALUE! | 16.47955 | #VALUE! | #VALUE! | 16.18495 | #VALUE! | #VALUE! | 17.436987 | #VALUE! | 19.224916 | #VALUE! | #VALUE! | #VALUE! | #VALUE! |
| 251 | hsa-miR-507-4373232 | #VALUE! | #VALUE! | #VALUE! | #VALUE! | #VALUE! | #VALUE! | #VALUE! | #VALUE! | #VALUE! | #VALUE! | #VALUE! | #VALUE! | #VALUE! | #VALUE! |
| 252 | hsa-miR-508-3p-4373233 | #VALUE! | #VALUE! | #VALUE! | 19.330142 | #VALUE! | #VALUE! | 19.330142 | #VALUE! | #VALUE! | #VALUE! | #VALUE! | #VALUE! | #VALUE! | #VALUE! |
| 253 | hsa-miR-508-5p-4395203 | #VALUE! | #VALUE! | #VALUE! | #VALUE! | #VALUE! | #VALUE! | #VALUE! | #VALUE! | #VALUE! | #VALUE! | #VALUE! | #VALUE! | #VALUE! | #VALUE! |
| 254 | hsa-miR-509-5p-4395346 | #VALUE! | #VALUE! | #VALUE! | 19.358123 | 16.096608 | #VALUE! | 19.358123 | 18.368174 | #VALUE! | #VALUE! | #VALUE! | #VALUE! | #VALUE! | #VALUE! |
| 255 | hsa-miR-510-4395352 | #VALUE! | #VALUE! | #VALUE! | #VALUE! | #VALUE! | #VALUE! | #VALUE! | #VALUE! | #VALUE! | #VALUE! | #VALUE! | #VALUE! | #VALUE! | #VALUE! |
| 256 | hsa-miR-512-3p-4381034 | #VALUE! | #VALUE! | #VALUE! | #VALUE! | 19.17688 | #VALUE! | #VALUE! | 19.397143 | #VALUE! | 17.290874 | 18.1672 | #VALUE! | #VALUE! | #VALUE! |
| 257 | hsa-miR-512-5p-4373238 | #VALUE! | #VALUE! | #VALUE! | #VALUE! | 19.13348 | #VALUE! | #VALUE! | #VALUE! | #VALUE! | #VALUE! | #VALUE! | #VALUE! | #VALUE! | #VALUE! |
| 258 | hsa-miR-513-5p-4395201 | #VALUE! | #VALUE! | #VALUE! | #VALUE! | #VALUE! | #VALUE! | #VALUE! | #VALUE! | #VALUE! | #VALUE! | #VALUE! | #VALUE! | #VALUE! | #VALUE! |
| 259 | hsa-miR-515-3p-4395480 | #VALUE! | #VALUE! | #VALUE! | #VALUE! | #VALUE! | #VALUE! | #VALUE! | #VALUE! | #VALUE! | 21.258084 | #VALUE! | #VALUE! | #VALUE! | #VALUE! |
| 260 | hsa-miR-515-5p-4373242 | #VALUE! | #VALUE! | #VALUE! | #VALUE! | #VALUE! | #VALUE! | #VALUE! | #VALUE! | #VALUE! | #VALUE! | #VALUE! | #VALUE! | #VALUE! | #VALUE! |
| 261 | hsa-miR-516a-5p-4395527 | #VALUE! | #VALUE! | #VALUE! | #VALUE! | #VALUE! | #VALUE! | #VALUE! | #VALUE! | #VALUE! | #VALUE! | #VALUE! | #VALUE! | #VALUE! | #VALUE! |
| 262 | hsa-miR-516b-4395172 | #VALUE! | #VALUE! | #VALUE! | #VALUE! | #VALUE! | #VALUE! | #VALUE! | #VALUE! | #VALUE! | #VALUE! | #VALUE! | #VALUE! | #VALUE! | #VALUE! |
| 263 | hsa-miR-517a-4395513 | 20.156676 | 19.444364 | #VALUE! | #VALUE! | #VALUE! | #VALUE! | #VALUE! | 19.414546 | 20.138364 | 18.27479 | 17.129326 | #VALUE! | #VALUE! | #VALUE! |
| 264 | hsa-miR-517c-4373264 | 21.11074 | #VALUE! | 18.2142 | #VALUE! | 19.112626 | #VALUE! | #VALUE! | #VALUE! | 19.085104 | 19.264905 | 17.118847 | #VALUE! | #VALUE! | #VALUE! |
| 265 | hsa-miR-518a-3p-4395508 | #VALUE! | #VALUE! | #VALUE! | #VALUE! | #VALUE! | #VALUE! | #VALUE! | #VALUE! | #VALUE! | 22.26688 | #VALUE! | #VALUE! | #VALUE! | #VALUE! |
| 266 | hsa-miR-518a-5p-4395507 | #VALUE! | #VALUE! | #VALUE! | #VALUE! | #VALUE! | #VALUE! | #VALUE! | #VALUE! | #VALUE! | #VALUE! | #VALUE! | #VALUE! | #VALUE! | #VALUE! |
| 267 | hsa-miR-518b-4373246 | 18.207072 | 17.433916 | 16.196517 | 19.33826 | 18.139317 | 17.8630164 | 19.33826 | 17.415576 | 20.107923 | 20.47134 | #VALUE! | #VALUE! | #VALUE! | #VALUE! |
| 268 | hsa-miR-518c-4395512 | #VALUE! | #VALUE! | #VALUE! | #VALUE! | #VALUE! | #VALUE! | #VALUE! | #VALUE! | #VALUE! | #VALUE! | #VALUE! | #VALUE! | #VALUE! | #VALUE! |
| 269 | hsa-miR-518d-3p-4373248 | 18.13238 | #VALUE! | #VALUE! | #VALUE! | #VALUE! | #VALUE! | #VALUE! | #VALUE! | #VALUE! | 21.278485 | #VALUE! | #VALUE! | #VALUE! | #VALUE! |
| 270 | hsa-miR-518d-5p-4395500 | #VALUE! | #VALUE! | #VALUE! | #VALUE! | #VALUE! | #VALUE! | #VALUE! | #VALUE! | #VALUE! | #VALUE! | #VALUE! | #VALUE! | #VALUE! | #VALUE! |
| 271 | hsa-miR-518e-4395506 | #VALUE! | 19.455183 | 17.315215 | 21.450603 | 19.163327 | #VALUE! | 21.450603 | 19.36417 | #VALUE! | 22.253804 | #VALUE! | #VALUE! | #VALUE! | #VALUE! |
| 272 | hsa-miR-518f-4395499 | #VALUE! | #VALUE! | 18.183357 | 22.295768 | #VALUE! | #VALUE! | 22.295768 | 19.37404 | 21.07186 | 22.234 | 17.076256 | 20.4103848 | #VALUE! | #VALUE! |
| 273 | hsa-miR-519a-4395526 | 21.15985 | 20.417398 | 16.276485 | #VALUE! | #VALUE! | #VALUE! | #VALUE! | #VALUE! | #VALUE! | 20.27185 | 19.15372 | #VALUE! | #VALUE! | #VALUE! |
| 274 | hsa-miR-519d-4395514 | 19.137503 | #VALUE! | #VALUE! | 20.317016 | 19.14576 | #VALUE! | 20.317016 | #VALUE! | #VALUE! | #VALUE! | 19.08225 | #VALUE! | #VALUE! | #VALUE! |
| 275 | hsa-miR-519e-4395481 | #VALUE! | #VALUE! | #VALUE! | #VALUE! | #VALUE! | #VALUE! | #VALUE! | #VALUE! | #VALUE! | #VALUE! | #VALUE! | #VALUE! | #VALUE! | #VALUE! |
| 276 | hsa-miR-520a-3p-4373268 | #VALUE! | #VALUE! | #VALUE! | #VALUE! | #VALUE! | #VALUE! | #VALUE! | #VALUE! | #VALUE! | #VALUE! | #VALUE! | #VALUE! | #VALUE! | #VALUE! |
| 277 | hsa-miR-520a-5p-4378085 | #VALUE! | #VALUE! | #VALUE! | #VALUE! | #VALUE! | #VALUE! | #VALUE! | #VALUE! | #VALUE! | #VALUE! | #VALUE! | #VALUE! | #VALUE! | #VALUE! |
| 278 | hsa-miR-520d-5p-4395504 | #VALUE! | #VALUE! | #VALUE! | #VALUE! | #VALUE! | #VALUE! | #VALUE! | #VALUE! | #VALUE! | #VALUE! | #VALUE! | #VALUE! | #VALUE! | #VALUE! |
| 279 | hsa-miR-520g-4373257 | #VALUE! | #VALUE! | #VALUE! | #VALUE! | #VALUE! | #VALUE! | #VALUE! | 19.412463 | 20.11061 | 21.2367 | #VALUE! | #VALUE! | #VALUE! | #VALUE! |
| 280 | hsa-miR-521-4373259 | #VALUE! | #VALUE! | #VALUE! | #VALUE! | #VALUE! | #VALUE! | #VALUE! | #VALUE! | #VALUE! | #VALUE! | #VALUE! | #VALUE! | #VALUE! | #VALUE! |
| 281 | hsa-miR-522-4395524 | 19.13033 | 19.403345 | 18.258025 | #VALUE! | #VALUE! | #VALUE! | #VALUE! | 18.4406 | 21.21331 | 21.273965 | #VALUE! | #VALUE! | #VALUE! | #VALUE! |
| 282 | hsa-miR-523-4395497 | 20.130122 | 20.489618 | #VALUE! | 22.163333 | 20.092057 | #VALUE! | 22.163333 | #VALUE! | #VALUE! | 22.23379 | 18.093586 | #VALUE! | #VALUE! | #VALUE! |
| 283 | hsa-miR-524-5p-4395174 | #VALUE! | #VALUE! | #VALUE! | #VALUE! | #VALUE! | #VALUE! | #VALUE! | #VALUE! | #VALUE! | #VALUE! | #VALUE! | #VALUE! | #VALUE! | #VALUE! |
| 284 | hsa-miR-525-3p-4395496 | 18.322665 | #VALUE! | #VALUE! | #VALUE! | #VALUE! | #VALUE! | #VALUE! | #VALUE! | #VALUE! | 19.36432 | #VALUE! | #VALUE! | #VALUE! | #VALUE! |
| 285 | hsa-miR-525-5p-4378088 | #VALUE! | #VALUE! | #VALUE! | #VALUE! | #VALUE! | #VALUE! | #VALUE! | #VALUE! | #VALUE! | #VALUE! | #VALUE! | #VALUE! | #VALUE! | #VALUE! |
| 286 | hsa-miR-526b-4395493 | #VALUE! | #VALUE! | #VALUE! | #VALUE! | #VALUE! | #VALUE! | #VALUE! | #VALUE! | #VALUE! | #VALUE! | #VALUE! | #VALUE! | #VALUE! | #VALUE! |
| 290 | hsa-miR-541-4395312 | 18.19689 | 17.514734 | #VALUE! | #VALUE! | #VALUE! | #VALUE! | #VALUE! | 18.40523 | #VALUE! | #VALUE! | #VALUE! | #VALUE! | #VALUE! | #VALUE! |
| 291 | hsa-miR-542-3p-4378101 | 21.142028 | 20.42777 | #VALUE! | 22.32865 | 19.188886 | #VALUE! | 22.32865 | 19.374686 | 21.04763 | 22.307595 | #VALUE! | #VALUE! | #VALUE! | #VALUE! |
| 293 | hsa-miR-544-4395376 | #VALUE! | #VALUE! | #VALUE! | #VALUE! | #VALUE! | #VALUE! | #VALUE! | #VALUE! | #VALUE! | #VALUE! | #VALUE! | #VALUE! | #VALUE! | #VALUE! |
| 294 | hsa-miR-545-4395378 | 19.123492 | 18.419908 | 18.222957 | 22.318706 | 17.136773 | 19.0443672 | 22.318706 | 19.35628 | 18.07311 | 21.25864 | #VALUE! | #VALUE! | #VALUE! | #VALUE! |
| 295 | hsa-miR-548a-3p-4380948 | #VALUE! | #VALUE! | #VALUE! | #VALUE! | #VALUE! | #VALUE! | #VALUE! | #VALUE! | #VALUE! | #VALUE! | #VALUE! | #VALUE! | #VALUE! | #VALUE! |
| 296 | hsa-miR-548a-5p-4395523 | #VALUE! | #VALUE! | #VALUE! | #VALUE! | #VALUE! | #VALUE! | #VALUE! | #VALUE! | #VALUE! | #VALUE! | #VALUE! | #VALUE! | #VALUE! | #VALUE! |
| 297 | hsa-miR-548b-3p-4380951 | #VALUE! | #VALUE! | #VALUE! | #VALUE! | #VALUE! | #VALUE! | #VALUE! | #VALUE! | #VALUE! | #VALUE! | #VALUE! | #VALUE! | #VALUE! | #VALUE! |
| 298 | hsa-miR-548b-5p-4395519 | 18.17658 | #VALUE! | #VALUE! | #VALUE! | 19.160817 | #VALUE! | #VALUE! | 19.37912 | 20.09541 | 20.290284 | 19.14428 | #VALUE! | #VALUE! | #VALUE! |
| 299 | hsa-miR-548c-3p-4380993 | #VALUE! | #VALUE! | #VALUE! | #VALUE! | #VALUE! | #VALUE! | #VALUE! | #VALUE! | #VALUE! | #VALUE! | #VALUE! | #VALUE! | #VALUE! | #VALUE! |
| 300 | hsa-miR-548c-5p-4395540 | #VALUE! | 20.387563 | 19.22968 | #VALUE! | #VALUE! | #VALUE! | #VALUE! | 20.350276 | #VALUE! | 22.232427 | 18.114003 | #VALUE! | #VALUE! | #VALUE! |
| 301 | hsa-miR-548d-3p-4381008 | #VALUE! | #VALUE! | #VALUE! | #VALUE! | 20.159177 | #VALUE! | #VALUE! | #VALUE! | #VALUE! | #VALUE! | #VALUE! | #VALUE! | #VALUE! | #VALUE! |
| 302 | hsa-miR-548d-5p-4395348 | 18.10852 | 19.48063 | 18.230197 | 21.369285 | #VALUE! | #VALUE! | 21.369285 | 20.40468 | 19.992105 | 21.312116 | #VALUE! | #VALUE! | #VALUE! | #VALUE! |
| 303 | hsa-miR-551b-4380945 | #VALUE! | #VALUE! | #VALUE! | #VALUE! | #VALUE! | #VALUE! | #VALUE! | #VALUE! | #VALUE! | #VALUE! | #VALUE! | #VALUE! | #VALUE! | #VALUE! |
| 304 | hsa-miR-556-3p-4395456 | #VALUE! | #VALUE! | #VALUE! | #VALUE! | #VALUE! | #VALUE! | #VALUE! | #VALUE! | #VALUE! | #VALUE! | #VALUE! | #VALUE! | #VALUE! | #VALUE! |
| 305 | hsa-miR-556-5p-4395455 | #VALUE! | #VALUE! | #VALUE! | #VALUE! | #VALUE! | #VALUE! | #VALUE! | #VALUE! | #VALUE! | #VALUE! | #VALUE! | #VALUE! | #VALUE! | #VALUE! |
| 306 | hsa-miR-561-4380938 | #VALUE! | #VALUE! | #VALUE! | #VALUE! | #VALUE! | #VALUE! | #VALUE! | #VALUE! | #VALUE! | #VALUE! | #VALUE! | #VALUE! | #VALUE! | #VALUE! |
| 307 | hsa-miR-570-4395458 | 20.162253 | #VALUE! | #VALUE! | 22.26399 | 19.192242 | #VALUE! | 22.26399 | #VALUE! | #VALUE! | 21.38396 | #VALUE! | #VALUE! | #VALUE! | #VALUE! |
| 309 | hsa-miR-576-3p-4395462 | 20.18315 | 18.431413 | 17.26542 | 21.30187 | 18.186444 | 19.0736594 | 21.30187 | 18.389987 | 20.112566 | 20.274057 | #VALUE! | #VALUE! | #VALUE! | #VALUE! |
| 310 | hsa-miR-576-5p-4395461 | 19.1011 | #VALUE! | #VALUE! | #VALUE! | #VALUE! | #VALUE! | #VALUE! | #VALUE! | #VALUE! | #VALUE! | #VALUE! | #VALUE! | #VALUE! | #VALUE! |
| 312 | hsa-miR-582-3p-4395510 | 20.11992 | #VALUE! | #VALUE! | #VALUE! | 20.235776 | #VALUE! | #VALUE! | #VALUE! | 21.107328 | 21.250413 | #VALUE! | #VALUE! | #VALUE! | #VALUE! |
| 313 | hsa-miR-582-5p-4395175 | 19.160964 | 19.454793 | #VALUE! | 21.305797 | 19.231522 | #VALUE! | 21.305797 | #VALUE! | 20.141363 | 19.304525 | #VALUE! | #VALUE! | #VALUE! | #VALUE! |
| 314 | hsa-miR-589-4395520 | #VALUE! | #VALUE! | #VALUE! | #VALUE! | 19.15472 | #VALUE! | #VALUE! | #VALUE! | #VALUE! | #VALUE! | #VALUE! | #VALUE! | #VALUE! | #VALUE! |
| 316 | hsa-miR-597-4380960 | 19.121253 | #VALUE! | 17.239333 | 21.287917 | 17.15225 | #VALUE! | 21.287917 | 19.388648 | 21.325296 | #VALUE! | 18.092728 | #VALUE! | #VALUE! | #VALUE! |
| 318 | hsa-miR-615-3p-4386777 | #VALUE! | #VALUE! | #VALUE! | #VALUE! | #VALUE! | #VALUE! | #VALUE! | #VALUE! | #VALUE! | #VALUE! | #VALUE! | #VALUE! | #VALUE! | #VALUE! |
| 319 | hsa-miR-615-5p-4395464 | #VALUE! | #VALUE! | #VALUE! | #VALUE! | #VALUE! | #VALUE! | #VALUE! | #VALUE! | #VALUE! | #VALUE! | #VALUE! | #VALUE! | #VALUE! | #VALUE! |
| 320 | hsa-miR-616-4395525 | #VALUE! | #VALUE! | #VALUE! | #VALUE! | #VALUE! | #VALUE! | #VALUE! | #VALUE! | 18.11222 | #VALUE! | #VALUE! | #VALUE! | #VALUE! | #VALUE! |
| 321 | hsa-miR-618-4380996 | 19.126414 | 20.411005 | 19.388503 | 22.087867 | 20.17984 | 20.2387258 | 22.087867 | #VALUE! | #VALUE! | 22.227456 | 18.11719 | #VALUE! | #VALUE! | #VALUE! |
| 322 | hsa-miR-624-4395541 | #VALUE! | #VALUE! | #VALUE! | #VALUE! | #VALUE! | #VALUE! | #VALUE! | #VALUE! | #VALUE! | #VALUE! | #VALUE! | #VALUE! | #VALUE! | #VALUE! |
| 326 | hsa-miR-629-4395547 | 20.142264 | 19.43822 | 18.24328 | #VALUE! | 19.196027 | #VALUE! | #VALUE! | 20.408988 | #VALUE! | 21.27111 | #VALUE! | #VALUE! | #VALUE! | #VALUE! |
| 328 | hsa-miR-642-4380995 | 19.125987 | 20.38663 | 17.268695 | #VALUE! | 17.14876 | #VALUE! | #VALUE! | 17.389956 | 19.118185 | 21.280156 | #VALUE! | #VALUE! | #VALUE! | #VALUE! |
| 329 | hsa-miR-651-4381007 | #VALUE! | #VALUE! | #VALUE! | #VALUE! | 18.181527 | #VALUE! | #VALUE! | #VALUE! | #VALUE! | #VALUE! | #VALUE! | #VALUE! | #VALUE! | #VALUE! |
| 331 | hsa-miR-653-4395403 | #VALUE! | #VALUE! | #VALUE! | #VALUE! | 19.14744 | #VALUE! | #VALUE! | #VALUE! | #VALUE! | #VALUE! | #VALUE! | #VALUE! | #VALUE! | #VALUE! |
| 332 | hsa-miR-654-3p-4395350 | 20.168002 | 19.407762 | #VALUE! | 21.27823 | 18.15642 | #VALUE! | 21.27823 | #VALUE! | #VALUE! | 20.272462 | #VALUE! | #VALUE! | #VALUE! | #VALUE! |
| 333 | hsa-miR-654-5p-4381014 | #VALUE! | #VALUE! | #VALUE! | #VALUE! | #VALUE! | #VALUE! | #VALUE! | #VALUE! | #VALUE! | #VALUE! | #VALUE! | #VALUE! | #VALUE! | #VALUE! |
| 336 | hsa-miR-671-3p-4395433 | 19.146548 | 17.461225 | 16.265205 | #VALUE! | 17.184182 | #VALUE! | #VALUE! | 18.398295 | 19.105978 | 20.30834 | #VALUE! | #VALUE! | #VALUE! | #VALUE! |
| 337 | hsa-miR-672-4395438 | #VALUE! | 19.41412 | #VALUE! | #VALUE! | #VALUE! | #VALUE! | #VALUE! | #VALUE! | 20.225694 | 21.292234 | #VALUE! | #VALUE! | #VALUE! | #VALUE! |
| 338 | hsa-miR-674-4395193 | #VALUE! | #VALUE! | #VALUE! | #VALUE! | #VALUE! | #VALUE! | #VALUE! | #VALUE! | #VALUE! | #VALUE! | #VALUE! | #VALUE! | #VALUE! | #VALUE! |
| 341 | hsa-miR-758-4395180 | 18.125556 | 17.419332 | #VALUE! | #VALUE! | 18.189435 | #VALUE! | #VALUE! | 19.41132 | 20.120813 | #VALUE! | 17.189705 | #VALUE! | #VALUE! | #VALUE! |
| 342 | hsa-miR-871-4395465 | #VALUE! | #VALUE! | #VALUE! | #VALUE! | #VALUE! | #VALUE! | #VALUE! | #VALUE! | #VALUE! | #VALUE! | #VALUE! | #VALUE! | #VALUE! | #VALUE! |
| 343 | hsa-miR-872-4395375 | #VALUE! | #VALUE! | #VALUE! | #VALUE! | #VALUE! | #VALUE! | #VALUE! | #VALUE! | #VALUE! | #VALUE! | #VALUE! | #VALUE! | #VALUE! | #VALUE! |
| 344 | hsa-miR-873-4395467 | #VALUE! | #VALUE! | #VALUE! | #VALUE! | #VALUE! | #VALUE! | #VALUE! | #VALUE! | #VALUE! | #VALUE! | #VALUE! | #VALUE! | #VALUE! | #VALUE! |
| 345 | hsa-miR-874-4395379 | #VALUE! | 13.421995 | #VALUE! | #VALUE! | #VALUE! | #VALUE! | #VALUE! | 13.35664 | #VALUE! | #VALUE! | #VALUE! | #VALUE! | #VALUE! | #VALUE! |
| 346 | hsa-miR-875-3p-4395315 | #VALUE! | #VALUE! | #VALUE! | #VALUE! | #VALUE! | #VALUE! | #VALUE! | #VALUE! | #VALUE! | #VALUE! | #VALUE! | #VALUE! | #VALUE! | #VALUE! |
| 347 | hsa-miR-876-3p-4395336 | #VALUE! | #VALUE! | #VALUE! | #VALUE! | #VALUE! | #VALUE! | #VALUE! | #VALUE! | #VALUE! | #VALUE! | #VALUE! | #VALUE! | #VALUE! | #VALUE! |
| 348 | hsa-miR-876-5p-4395316 | #VALUE! | #VALUE! | #VALUE! | #VALUE! | #VALUE! | #VALUE! | #VALUE! | #VALUE! | #VALUE! | #VALUE! | #VALUE! | #VALUE! | #VALUE! | #VALUE! |
| 349 | hsa-miR-885-3p-4395483 | #VALUE! | #VALUE! | #VALUE! | #VALUE! | #VALUE! | #VALUE! | #VALUE! | #VALUE! | #VALUE! | #VALUE! | #VALUE! | #VALUE! | #VALUE! | #VALUE! |
| 353 | hsa-miR-887-4395485 | #VALUE! | #VALUE! | #VALUE! | #VALUE! | #VALUE! | #VALUE! | #VALUE! | #VALUE! | #VALUE! | #VALUE! | #VALUE! | #VALUE! | #VALUE! | #VALUE! |
| 354 | hsa-miR-888-4395323 | #VALUE! | #VALUE! | 18.275073 | #VALUE! | 19.171937 | #VALUE! | #VALUE! | #VALUE! | #VALUE! | #VALUE! | #VALUE! | #VALUE! | #VALUE! | #VALUE! |
| 355 | hsa-miR-889-4395313 | 19.12376 | 19.47644 | 17.250137 | #VALUE! | 18.181046 | #VALUE! | #VALUE! | 18.42318 | #VALUE! | 20.295606 | 18.13743 | #VALUE! | #VALUE! | #VALUE! |
| 356 | hsa-miR-890-4395320 | #VALUE! | #VALUE! | #VALUE! | #VALUE! | #VALUE! | #VALUE! | #VALUE! | #VALUE! | #VALUE! | #VALUE! | #VALUE! | #VALUE! | #VALUE! | #VALUE! |
| 357 | hsa-miR-891a-4395302 | 20.052554 | 19.462987 | 18.226363 | #VALUE! | #VALUE! | #VALUE! | #VALUE! | #VALUE! | #VALUE! | #VALUE! | #VALUE! | #VALUE! | #VALUE! | #VALUE! |
| 358 | hsa-miR-891b-4395321 | #VALUE! | #VALUE! | #VALUE! | #VALUE! | #VALUE! | #VALUE! | #VALUE! | #VALUE! | #VALUE! | #VALUE! | #VALUE! | #VALUE! | #VALUE! | #VALUE! |
| 359 | hsa-miR-892a-4395306 | #VALUE! | #VALUE! | #VALUE! | #VALUE! | #VALUE! | #VALUE! | #VALUE! | #VALUE! | #VALUE! | #VALUE! | #VALUE! | #VALUE! | #VALUE! | #VALUE! |
| 360 | hsa-miR-147-4373131 | #VALUE! | #VALUE! | #VALUE! | #VALUE! | #VALUE! | #VALUE! | #VALUE! | #VALUE! | #VALUE! | #VALUE! | #VALUE! | #VALUE! | #VALUE! | #VALUE! |
| 361 | hsa-miR-208-4373091 | #VALUE! | #VALUE! | #VALUE! | #VALUE! | #VALUE! | #VALUE! | #VALUE! | #VALUE! | #VALUE! | #VALUE! | #VALUE! | #VALUE! | #VALUE! | #VALUE! |
| 362 | hsa-miR-211-4373088 | #VALUE! | #VALUE! | #VALUE! | #VALUE! | #VALUE! | #VALUE! | #VALUE! | #VALUE! | #VALUE! | #VALUE! | 18.104844 | #VALUE! | #VALUE! | #VALUE! |
| 364 | hsa-miR-219-1-3p-4395206 | #VALUE! | #VALUE! | #VALUE! | #VALUE! | 19.13697 | #VALUE! | #VALUE! | #VALUE! | #VALUE! | #VALUE! | #VALUE! | #VALUE! | #VALUE! | #VALUE! |
| 365 | hsa-miR-219-2-3p-4395501 | #VALUE! | #VALUE! | #VALUE! | #VALUE! | #VALUE! | #VALUE! | #VALUE! | #VALUE! | #VALUE! | #VALUE! | #VALUE! | #VALUE! | #VALUE! | #VALUE! |
| 366 | hsa-miR-220-4373078 | #VALUE! | #VALUE! | #VALUE! | #VALUE! | #VALUE! | #VALUE! | #VALUE! | #VALUE! | #VALUE! | #VALUE! | #VALUE! | #VALUE! | #VALUE! | #VALUE! |
| 367 | hsa-miR-220b-4395317 | #VALUE! | #VALUE! | #VALUE! | #VALUE! | #VALUE! | #VALUE! | #VALUE! | #VALUE! | #VALUE! | #VALUE! | #VALUE! | #VALUE! | #VALUE! | #VALUE! |
| 368 | hsa-miR-220c-4395322 | #VALUE! | #VALUE! | #VALUE! | #VALUE! | #VALUE! | #VALUE! | #VALUE! | #VALUE! | #VALUE! | #VALUE! | #VALUE! | #VALUE! | #VALUE! | #VALUE! |
| 369 | hsa-miR-298-4395301 | #VALUE! | #VALUE! | #VALUE! | #VALUE! | #VALUE! | #VALUE! | #VALUE! | #VALUE! | #VALUE! | #VALUE! | #VALUE! | #VALUE! | #VALUE! | #VALUE! |
| 370 | hsa-miR-325-4373051 | #VALUE! | #VALUE! | #VALUE! | #VALUE! | #VALUE! | #VALUE! | #VALUE! | #VALUE! | #VALUE! | #VALUE! | #VALUE! | #VALUE! | #VALUE! | #VALUE! |
| 371 | hsa-miR-346-4373038 | #VALUE! | #VALUE! | #VALUE! | #VALUE! | #VALUE! | #VALUE! | #VALUE! | #VALUE! | #VALUE! | #VALUE! | #VALUE! | #VALUE! | #VALUE! | #VALUE! |
| 373 | hsa-miR-384-4373017 | #VALUE! | #VALUE! | #VALUE! | #VALUE! | #VALUE! | #VALUE! | #VALUE! | #VALUE! | #VALUE! | #VALUE! | #VALUE! | #VALUE! | #VALUE! | #VALUE! |
| 374 | hsa-miR-412-4373199 | 17.18342 | #VALUE! | #VALUE! | #VALUE! | #VALUE! | #VALUE! | #VALUE! | #VALUE! | #VALUE! | #VALUE! | #VALUE! | #VALUE! | #VALUE! | #VALUE! |
| 375 | hsa-miR-448-4373206 | #VALUE! | #VALUE! | #VALUE! | #VALUE! | #VALUE! | #VALUE! | #VALUE! | #VALUE! | #VALUE! | #VALUE! | #VALUE! | #VALUE! | #VALUE! | #VALUE! |
| 376 | hsa-miR-492-4373217 | #VALUE! | #VALUE! | #VALUE! | #VALUE! | #VALUE! | #VALUE! | #VALUE! | 14.387059 | 17.175638 | #VALUE! | #VALUE! | #VALUE! | #VALUE! | #VALUE! |
| 377 | hsa-miR-506-4373231 | #VALUE! | #VALUE! | #VALUE! | #VALUE! | #VALUE! | #VALUE! | #VALUE! | #VALUE! | #VALUE! | #VALUE! | #VALUE! | #VALUE! | #VALUE! | #VALUE! |
| 378 | hsa-miR-509-3-5p-4395266 | #VALUE! | #VALUE! | #VALUE! | #VALUE! | #VALUE! | #VALUE! | #VALUE! | #VALUE! | #VALUE! | #VALUE! | #VALUE! | #VALUE! | #VALUE! | #VALUE! |
| 380 | hsa-miR-517b-4373244 | #VALUE! | #VALUE! | #VALUE! | #VALUE! | #VALUE! | #VALUE! | #VALUE! | #VALUE! | #VALUE! | #VALUE! | #VALUE! | #VALUE! | #VALUE! | #VALUE! |
| 381 | hsa-miR-519c-3p-4373251 | #VALUE! | #VALUE! | #VALUE! | #VALUE! | #VALUE! | #VALUE! | #VALUE! | #VALUE! | #VALUE! | #VALUE! | #VALUE! | #VALUE! | #VALUE! | #VALUE! |
| 382 | hsa-miR-520b-4373252 | #VALUE! | #VALUE! | #VALUE! | #VALUE! | #VALUE! | #VALUE! | #VALUE! | #VALUE! | #VALUE! | 22.360807 | #VALUE! | #VALUE! | #VALUE! | #VALUE! |
| 383 | hsa-miR-520e-4373255 | #VALUE! | #VALUE! | #VALUE! | #VALUE! | #VALUE! | #VALUE! | #VALUE! | #VALUE! | #VALUE! | #VALUE! | #VALUE! | #VALUE! | #VALUE! | #VALUE! |
| 384 | hsa-miR-520f-4373256 | #VALUE! | #VALUE! | #VALUE! | #VALUE! | 20.313756 | #VALUE! | #VALUE! | #VALUE! | #VALUE! | #VALUE! | #VALUE! | #VALUE! | #VALUE! | #VALUE! |
